# Creating High-Quality Synthetic Health Data: Framework for Model Development and Validation

**DOI:** 10.2196/53241

**Published:** 2024-04-22

**Authors:** Elnaz Karimian Sichani, Aaron Smith, Khaled El Emam, Lucy Mosquera

**Affiliations:** 1 Department of Mathematics and Statistics University of Ottawa Ottawa, ON Canada; 2 Children's Hospital of Eastern Ontario Research Institute Ottawa, ON Canada; 3 Replica Analytics Ltd Ottawa, ON Canada; 4 School of Epidemiology and Public Health University of Ottawa Ottawa, ON Canada

**Keywords:** synthetic data, tensor decomposition, data sharing, data utility, data privacy, electronic health record, longitudinal, model development, model validation, generative models

## Abstract

**Background:**

Electronic health records are a valuable source of patient information that must be properly deidentified before being shared with researchers. This process requires expertise and time. In addition, synthetic data have considerably reduced the restrictions on the use and sharing of real data, allowing researchers to access it more rapidly with far fewer privacy constraints. Therefore, there has been a growing interest in establishing a method to generate synthetic data that protects patients’ privacy while properly reflecting the data.

**Objective:**

This study aims to develop and validate a model that generates valuable synthetic longitudinal health data while protecting the privacy of the patients whose data are collected.

**Methods:**

We investigated the best model for generating synthetic health data, with a focus on longitudinal observations. We developed a generative model that relies on the generalized canonical polyadic (GCP) tensor decomposition. This model also involves sampling from a latent factor matrix of GCP decomposition, which contains patient factors, using sequential decision trees, copula, and Hamiltonian Monte Carlo methods. We applied the proposed model to samples from the MIMIC-III (version 1.4) data set. Numerous analyses and experiments were conducted with different data structures and scenarios. We assessed the similarity between our synthetic data and the real data by conducting utility assessments. These assessments evaluate the structure and general patterns present in the data, such as dependency structure, descriptive statistics, and marginal distributions. Regarding privacy disclosure, our model preserves privacy by preventing the direct sharing of patient information and eliminating the one-to-one link between the observed and model tensor records. This was achieved by simulating and modeling a latent factor matrix of GCP decomposition associated with patients.

**Results:**

The findings show that our model is a promising method for generating synthetic longitudinal health data that is similar enough to real data. It can preserve the utility and privacy of the original data while also handling various data structures and scenarios. In certain experiments, all simulation methods used in the model produced the same high level of performance. Our model is also capable of addressing the challenge of sampling patients from electronic health records. This means that we can simulate a variety of patients in the synthetic data set, which may differ in number from the patients in the original data.

**Conclusions:**

We have presented a generative model for producing synthetic longitudinal health data. The model is formulated by applying the GCP tensor decomposition. We have provided 3 approaches for the synthesis and simulation of a latent factor matrix following the process of factorization. In brief, we have reduced the challenge of synthesizing massive longitudinal health data to synthesizing a nonlongitudinal and significantly smaller data set.

## Introduction

### Background

Electronic health records (EHRs) are becoming an increasingly important source of detailed information about patients because the successful integration and efficient analysis of EHRs could help solve many health care problems, such as expediting clinical decisions and enhancing patient safety. However, researchers often encounter challenges when trying to obtain high-quality health data for their research, and EHRs need to be appropriately deidentified before being shared with researchers. This process requires both skill and effort.

A method for deidentifying data, including health data, is anonymization. However, recent allegations show successful reidentification attacks on anonymized data [1,2]. Data synthesis is a recently developed approach for data deidentification. Studies suggest that synthetic data do not pose a significant privacy risk [3,4]. Therefore, data synthesis has emerged as an interesting method for producing nonidentifiable health data, which reduces the restrictions on the use and sharing of actual data. This allows researchers to access it more rapidly and with substantially fewer privacy constraints.

Therefore, there has been growing interest in establishing a method to simulate synthetic data that protects patients’ privacy while properly reflecting the data. One method for generating synthetic data is to choose an appropriate model, fit it within privacy constraints, and then simulate new data from the fitted model.

Furthermore, dimensionality reduction converts the high-dimensional description of the data into a low dimension without losing crucial information and phenotypes [5]. In the medical context, phenotypes are used to describe relevant variations and features [6]. EHR-based phenotyping is a process that maps raw EHR data to meaningful medical concepts [7,8]. Moreover, synthesizing longitudinal data in EHRs becomes difficult because patients may have lengthy sequences of events and come from diverse populations. Therefore, using a dimensionality reduction technique in the generative model could be advantageous. However, the main challenge is to develop a model that captures the most important data features and efficiently finds meaningful characterizations.

Classical phenotyping methods, which require medical field experts, can be time-consuming and expensive [9,10] because of the high-dimensional and heterogeneous nature of the EHR data set. Recently, more efficient unsupervised approaches, such as matrix factorization [11], have been emerging. However, matrix factorization does not necessarily detect associations within a data set because the data may not be accurately represented as matrices. Thus, tensor decompositions [12] have drawn growing attention because of their interpretability and flexibility in accommodating high-dimensional data. They also appear to be beneficial and favorable for computational phenotyping [13] and can easily be privatized [14,15]. They have been recognized as a promising method for the analysis of EHRs [16]. Therefore, generating a model based on state-of-the-art tensor factorization for creating synthetic longitudinal health data is a valuable effort.

The goal of this study is to develop a model that generates valuable synthetic longitudinal health data while protecting the privacy of the patients whose data are collected. We propose a model that relies on generalized canonical polyadic (GCP) tensor decomposition, which has demonstrated substantial outcomes in health data analysis [17,18]. We validated this model using samples from the MIMIC-III (version 1.4) data set [19]. MIMIC-III is a large, freely available database that contains deidentified health-related data.

There is a widespread belief that synthetic data present an insignificant privacy risk because there is no unique link or mapping between the records in the synthetic data and the records in the original data [20]. Therefore, this view should also be satisfied in practice. However, there is a one-to-one mapping and direct correspondence between the entries of the GCP model and the entries of the original data. This undermines the most sensible and acceptable concepts of privacy. Inspired by the studies by Ma et al [14] and Schmidt and Mohamed [21], this is fixed by simulating and modeling a latent factor matrix of GCP decomposition associated with patients.

### Related Work

Tensor decomposition is an active area of research that has been widely applied to health care data [17,18,22]. It has been found to be an efficient method for phenotyping EHRs [13,23]. It also has various applications beyond health data analysis, including recommender systems [24] and signal processing [25]. Thus far, several tensor decomposition techniques have been developed [26]. The most popular technique is called canonical polyadic (CP) tensor factorization, which is also known by 2 different names: canonical tensor decomposition and parallel factor decomposition, which were introduced separately by Carroll and Chang [27] and Harshman [28], respectively.

The CP decomposition approximates a tensor by the sum of rank-1 tensors using squared errors (L2 loss) [26]. Recently, Hong et al [29] developed a GCP model that offers the flexibility to use other loss functions in addition to squared errors. Furthermore, Kolda and Hong [30] have presented stochastic gradient descent as a way to overcome the challenge of fitting generalized CP to large-scale tensors, making it a perfect fit for a massive and heterogeneous EHR data set.

CP factorization [28] and its generalization, GCP decomposition [29], are fundamental tools for tensor analysis. They result in a factorized tensor that contains the most important computational phenotypes, and many studies show that they perform particularly well in phenotyping EHRs [13,17]. In addition, privacy-preserving methods have been widely applied to them in the medical setting [14,15]. Therefore, we developed a generative model that produces synthetic longitudinal health data using generalized CP decomposition, which captures the most important features.

## Methods

In this section, we present the methods that we used to generate synthetic longitudinal health data using generalized CP decomposition and various sampling and simulation techniques. We also describe the data, the evaluation method, and the experimental details of our study.

### Notations and Definitions

We first describe the preliminaries and notations used in this paper. Before we begin, Table 1 shows some basic symbols used for tensor factorization. In addition, a boldface uppercase letter in Euler font represents a tensor, for example, 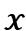. A matrix is represented by a boldface uppercase letter, such as **A**. A boldface lowercase letter, such as **a**, symbolizes a vector. A lowercase letter, such as *x*, denotes a scalar.

**Figure 1:**
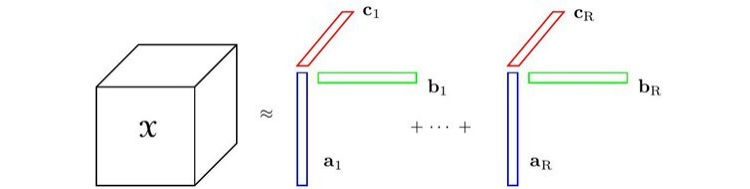
The generalized canonical polyadic decomposition.

The GCP decomposition approximates a *N*-way observed tensor 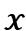 of size *n_1_ × n_2_ × ... × n_N_* by the sum of *R* rank-1 tensors as model 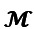, where *R* is smaller or equal to the rank of tensor 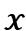, as illustrated in Figure 1. It is presented as follows [29]:



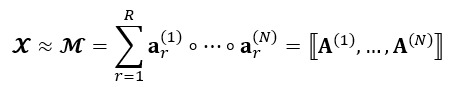



**(1)**

Where 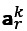 indicates the *r*th column of **A**^(*k*)^ for all *k*=1,...,*N* and *r=1,...,R*, and **A**^(*k*)^ is the *k*-mode factor matrix of size *I_k_× R, k=1,...,N,* consisting of *R* latent components or phenotypes vectors, expressed as follows:



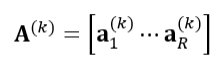



**(2)**

In addition, it is often convenient to express the decomposition with a positive weights vector of **λ** as follows:



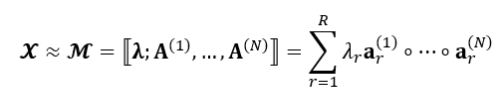



**(3)**

The GCP decomposition was carried out by minimizing the loss between 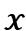 and 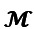 as represented by an objective function, which is also called a loss function. This means that finding factor matrices 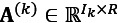 for *k = 1,...,N,* with a given *R* such that solves the following optimization problem:



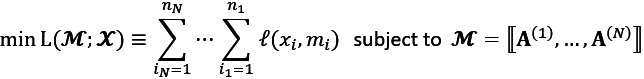



**(4)**

Where *x_i_ = x(i_1_,...,i_N_), m_i_ = m(i_1_,...,i_N_)*, and ℓ is the loss function. The choice of the loss function depends on how the original data are generated, which can be found in section S1 in Multimedia Appendix 1 [29].

Therefore, for a 3-way tensor 
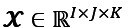
, the generalized CP decomposition with weight vector **λ**=**1** is represented as (for simplicity, **A**, **B**, **C** notations are used rather than **A**^(1)^, **A**^(2)^, **A**^(3)^):



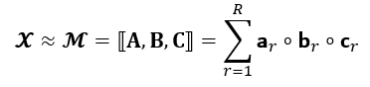



**(5)**

Where 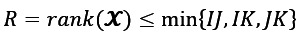, and 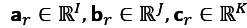 are the *r*th column vectors within the patient factor matrix 
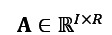
, and nonpatient factor matrices 
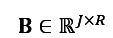
 and 
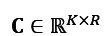
, respectively.

For simplicity, we discussed a 3-way tensor scenario; however, this approach generalizes to *N* modes as well. The GCP decomposes the observed longitudinal health data 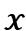 into 3-factor matrices: a patient factor matrix **A** and 2 nonpatient factor matrices **B** and **C**.

**Table 1:** The notations used in this paper.

Notations	Descriptions
◦	Outer product
*N*	Number of dimensions (modes) of a tensor
*R*	Number of ranks

### Data Synthesis Method

#### Overview

Our goal was to generate synthetic longitudinal health data, which we will refer to as 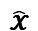. As mentioned earlier, relying on the model tensor 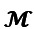 for synthesis is generally a bad idea and unwise because its elements directly approximate elements of the actual tensor 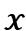 and thus disclosing privacy. Instead, we focused on the patient factor matrix **A** in the model’s latent space, which contains key phenotypes and information about patients.

**Figure 2:**
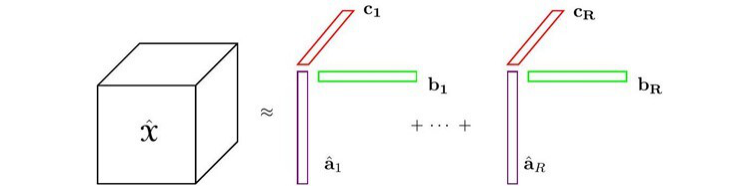
The generative model in terms of the generalized canonical polyadic decomposition.

In addition, the patient factor matrix **A** is not a longitudinal data set and is a significantly smaller data set than the model tensor 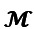; hence, its sampling and synthesis would be considerably simpler, and more efficient. Therefore, the aim was to find the optimal sampling or synthesis technique for the patient factor matrix **A**, which is denoted by 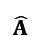. As determined by our research and study, we may use one of the generative models listed in the following subsections to create 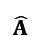. Thus, 
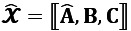
 is viewed as a synthetic form of 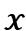, as illustrated in Figure 2:



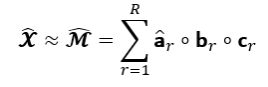



**(6)**

For addressing missing observations in the GCP model, Hong et al [29] used an indicator for the missingness of observations by assigning weights 0 if an observation is missing or 1 if it is observed; this approach for handling missing data is essentially the same as the work of Acar et al [31]. In addition, the missing structure of synthetic data must be similar to that of the original data. We assigned weights 0 or 1 to every element of the tensor 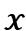, *x_i_*:



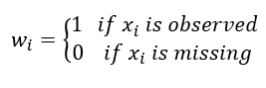



**(7)**

Let 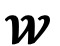 be the missingness indicator or mask tensor made of weights *w_i_* we then treated it as an input tensor and decomposed it using GCP decomposition. Assume 
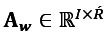
 is its patient factor matrix attained from the GCP decomposition with rank *R'*:



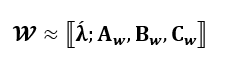



**(8)**

We proposed combining **A_w_** and **A** to create a new patient factor matrix 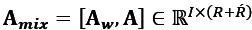, from which we sample and simulate to obtain the synthesized and simulated form of 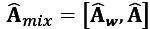. Then, we need to restore 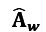 and 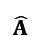
to their source latent space to obtain the synthetic data and its missingness indicator tensor 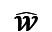.

Furthermore, the EHR data might be an irregular tensor, with patients having a varied number of clinical visits. However, the input for CP and generalized CP decompositions must be a regular tensor. Therefore, we proposed converting the irregular observed tensor into the regular one by adding extra missing visits. We then performed the GCP decomposition and added the number of clinical visits as a new variable to the patient factor matrix **A** because this feature is not a longitudinal variable and can be directly added to the patient factor matrix. We then sampled or synthesized the **A** along with that variable, and obtained the synthetic number of records, and finally, we modified the number of records in the postprocessing. We also recommended applying the same to the baseline attributes. With this generative model, we can simulate different numbers of patients in synthetic data as well.

#### Copula

This section summarizes the copula principles that we used in this study. Synthetic data generation using copula has recently attracted a lot of attention because some deep learning generative models, such as generative adversarial networks (GANs), require a very large data set for the learning stage and are therefore unproductive for small data sets. Copula models might be the most effective method to describe dependencies and marginal distributions. Furthermore, these models appear to be among the best options for data synthesis based on complex and small actual data sets [32]. Therefore, we selected the Gaussian copula as one of the simulation and synthesizing techniques for the patient factor matrix 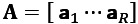 as it has been represented in several papers [33,34].

We assumed that 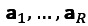 have marginal distributions *F_1_,...,F_R_* parametric or nonparametric, with covariances 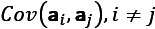. By a Gaussian copula, we completed the following:

1. Generated variables **t**_1_,...,**t**_R_ from a multivariate normal distribution with means all equal to 0, variances all equal to 1, and 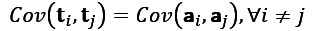.
2. Generated the uniform variables such that 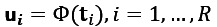; the Gaussian copula is the joint distribution of **u**_1_,...,**u**_R_
3. Generated samples 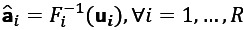.

However, the sampling will never be exact, and because we were sampling in the latent space, the correlation of the synthetic tensor might not be what we want. Therefore, we suggest selecting a sample such that the Frobenius norm of the difference between the correlation matrices of the original and synthetic data is less than a threshold ∈, which can be viewed as an optimization.

Finally, we produced the synthetic patient factor matrix from the obtained samples, such that 
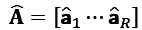
.

#### Sequential Decision Trees

Another technique that we proposed is to synthesize the patient factor matrix **A** using sequential decision trees, as presented in [35]. In data synthesis, sequential trees outperform deep learning methods, such as GANs or recurrent neural network models when the data set is not huge. We briefly summarize the sequential data synthesis process of [35]. Let us assume that the variables in the patient factor matrix **A** are 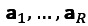, and their corresponding synthesized versions are 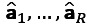. We fit *R-1* models using a model *M_j_* defined by 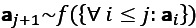, where *M_j_* and *f* denote a decision tree model and the tree model fitting function, respectively. Finally, we sampled 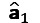 from **a**_1_ and applied 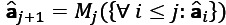 to sample the remaining variables.

#### Hamiltonian Monte Carlo

In this section, we sampled from the patient factor matrix **A** using a Bayesian simulation, with a focus on Hamiltonian Monte Carlo (HMC), which has advantages over other Markov Chain Monte Carlo (MCMC) methods.

We began by assuming that the patient factor matrix variables 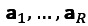 are generated from a multivariate Gaussian distribution prior with a known mean vector 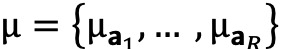. (which is set to a vector of zeros for simplicity) and an unknown covariance matrix ∑. A suitable prior for variances would be the Cauchy distribution. However, we must consider that the distribution of patient factor variables depends on the kind of loss function used in the GCP decomposition. We used this method only on the latent space generated by Gaussian loss.

The model block of STAN is provided in section S2 in Multimedia Appendix 1. In this block, *x* and *x_sim* represent the patient factor matrix variables **a** *_i_* and the corresponding simulations 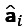, respectively. **N** indicates the number of patients.

### Data

This study used the MIMIC-III data set [19]. MIMIC-III is a large, freely available database that contains deidentified health-related data corresponding to >40,000 patients who stayed in critical care units. It was compiled at the Beth Israel Deaconess Medical Center in Boston, Massachusetts, between 2001 and 2012. Therefore, as the largest publicly available EHR data set, it has received significant interest from researchers as an open platform for the development and validation of their research on EHRs.

The continuous data set that we derived from the MIMIC-III data and used to evaluate the performance of our proposed model is a 3-way tensor of laboratory measurements for patients within the hospital who had 36 clinical visits. This is derived from the “LABEVENT” table in MIMIC-III. The resulting tensor consists of 226 patients, 4 laboratory tests (creatinine, potassium, sodium, and the hematocrit), and 36 clinical visits, with 21% of the data missing. The categorical data set used in our analysis is a tensor consisting of 246 patients, 2 categorical features (admission type and admission location), and 5 clinical visits, with no missing entries. This was obtained from the “ADMISSIONS” table of MIMIC-III. The detailed descriptions of the tables can be found in the study by Johnson et al [19].

### Evaluation Methods

We describe how we evaluated the utility and privacy risks of the synthetic data set.

The analysis of synthetic data should provide similar statistical inferences and conclusions to those obtained from actual data analysis. Therefore, we evaluated the proposed method on the utility aspect of the generated data [20,36].

We assessed the ability of the developed generalized CP framework to create synthetic data in terms of dependency structure and marginal fitting (univariate distribution similarity) using the following:

- The absolute difference in correlations between variables in the original and synthetic data
- The Hellinger distance between the synthetic and original variables indicates whether they are drawn from the same distribution. The Hellinger distance is a metric in the range of 0 to 1, where 0 indicates no difference between the distributions.
- The root mean square difference between the actual and synthetic correlations (RMSDC), which means the root mean square difference between the correlations of the original variables and those of the associated synthetic variables, is used to measure how well the dependency structure is captured. A lower RMSDC indicates a better capture of the dependency structure.
- Descriptive statistics

The statistical characteristics of a synthetic data set need to match those of the original data. However, a single record in the synthetic data does not relate to or correspond to a single record in the original data set [13]. There is a one-to-one correspondence between the entries of the GCP model and the original data, which violates the most reasonable and accepted concepts of privacy. Therefore, in terms of privacy disclosure, our approach preserves privacy by preventing the direct sharing of patient information and destroying the one-to-one link between the observed and model tensor records.

### Experimental Details

We evaluated 3 different simulation and sampling techniques on the generalized CP’s patient factor matrix, which contains patient phenotypes. Initially, we arranged the study into trials on dense and scarce continuous data sets and experiments on dense categorical data. Therefore, we started imputing the continuous data set with 21% missing observations using the GCP decomposition. We then used all 3 previously mentioned techniques to synthesize the imputed version of the original tensor, as well as the imputed data containing just the first 5 or 10 clinical visits.

The GCP decomposition was conducted using various loss functions depending on the kind of variables in our trials. These included gamma, β-divergence (similar to gamma), and Gaussian with nonnegativity constraints. Despite the continuous data set being nonnegative, the Gaussian loss (L2 loss) outperformed the others for all 3 approaches. This is because the dependency structure and univariate distributions of the original variables were significantly better preserved in the generated synthetic data. Refer to section S1 in Multimedia Appendix 1 for the choices of the loss function.

Finding the rank of a tensor is necessary for GCP decomposition. Following the selection of the loss function, we attempted to find the rank *R* by doing several runs with different values of *R*, where R ≤ min{*IJ*,*IK*,*JK*}, keeping in mind that the maximum rank is the last option. We then selected the rank for which there were no significant changes in the objective function, fit score 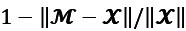, or mean square error from that rank to higher ranks. This allows important features and phenotypes to be captured by the model. This method is similar to the “elbow” rule. Although we may end up with the maximum value for *R*, the privacy constraints are not violated because the latent space simulation prevents overfitting. In addition, we investigated if the latent space structure, such as normalization and standardization, would lead to better results.

The GCP tensor decomposition was conducted using the algorithm of Hong et al [29], which was implemented in the Tensor Toolbox for MATLAB [37]. There is also a C++ programming software for GCP decompositions developed by Sandia National Laboratories [38], known as Genten, which is accessible in GitLab [39].

We applied the copula method using parametric and nonparametric marginals. For the nonparametric marginals, we used the empirical cumulative distribution function (CDF) and kernel smoothing. In addition, we performed the Gaussian copula based on parametric marginals using gamma, beta, and truncated Gaussian distributions, as suggested in the study by Benali et al [32].

We successfully synthesized the patient factor matrix **A** based on the sequential decision trees [35] through Replica Synthesis software [40]. The STAN model and the *rstan* package in R were used to perform HMC sampling on the patient factor matrix.

Finally, we applied sequential trees to validate the generative model on the data set with missing and irregular clinical visits. The categorical data trial was accomplished by GCP decomposition using Poisson with log link and Gaussian losses. Following that, the patient factor matrix was sampled using all 3 approaches.

### Ethical Considerations

This study was approved by the Children’s Hospital of Eastern Ontario (CHEO) Research Institute Research Ethics Board (protocol number 24/18X). The MIMIC-III database is a third-party anonymous public database approved by the Institutional Review Boards of Beth Israel Deaconess Medical Center (Boston, Massachusetts) and the Massachusetts Institute of Technology (Cambridge, Massachusetts).

## Results

### Overview

In this section, we report and analyze the results of our experiments on generating synthetic longitudinal health data using the proposed model. We demonstrate that our method is capable of handling different data structures and scenarios. We conducted numerous experiments and considered the following structures for both the original data and the synthetic data to validate our model:

- Synthetic data for dense original data with continuous variables
- Synthetic data with a varying number of patients compared to the original data
- Synthetic data for original data with missing observations
- Synthetic data for original data with irregular clinical visits
- Synthetic data for dense original data with categorical variables

### Experiments on Continuous Dense Data

According to the evaluations, β-divergence is not a suitable objective function for synthesizing continuous data in EHR with patient factor matrix simulation using sequential trees or HMC with the multivariate Gaussian distribution model. Refer to section S3 in Multimedia Appendix 1 for a summary of the findings.

We learned through several analyses that standardizing data and using Gaussian loss improves the results significantly. In the following, we present the outcomes of synthesizing a dense continuous data set that contains 226 patients, 4 laboratory test variables, and 5 clinical visits. In the preceding sections, we described how we obtained the data set for our study. The model used GCP decomposition with Gaussian loss and *R*=20. It can be observed that synthetic data sets generated by all 3 patient factor matrix simulation methods have comparable dependency structures and marginal distributions to the real ones. Here, we present the results of copula, sequential trees, and the HMC.

**Figure 3:**
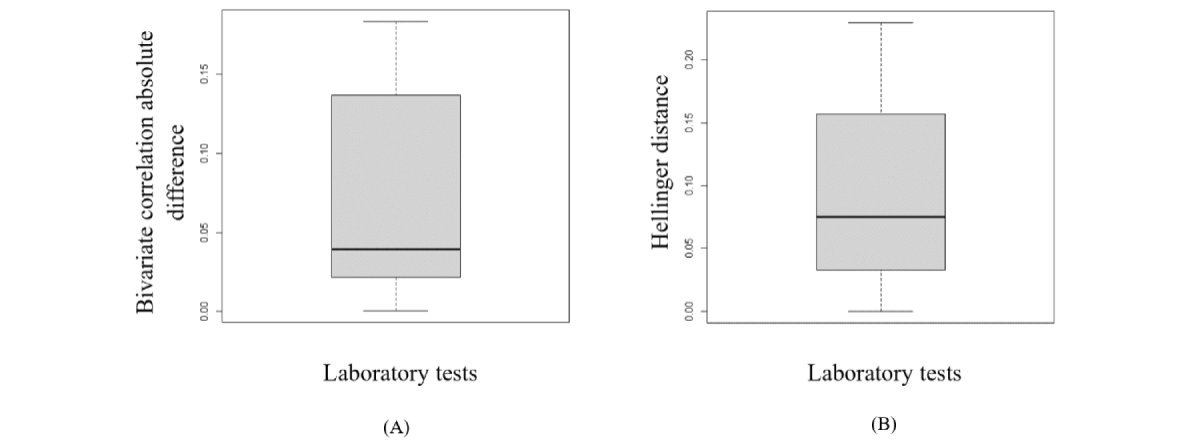
The box plots display the variation of the Hellinger distance and the Pearson correlation between the original variables and the synthetic variables generated by copula. (A) This is the box plot of the absolute differences in bivariate correlations between the real and synthetic data. Smaller values indicate that the bivariate relationships in the data have been greatly preserved during the generation of synthetic data. (B) This is the box plot of the Hellinger distance between the original variables and the synthetic variables. This shows the similarity of the univariate distributions between the real and synthetic data. This is a value between 0 and 1, with lower values indicating similarity between the univariate distributions of the real and synthetic variables.

According to section S4 in Multimedia Appendix 1, the synthetic data generated from the copula using empirical CDF marginals almost preserves the dependency structure and distribution of the original variables. The box plots in Figure 3 represent the variation in the Hellinger distance and the Pearson correlation between the variables in both the synthetic and original data sets. Furthermore, an RMSDC of 0.04 was obtained.

According to the summaries presented in Tables 2 and 3, the maximum value of the variables is slightly lower than that of the original.

The following are the outcomes of sampling the patient factor matrix of the previously mentioned GCP decomposition using the sequential trees approach. According to section S5 in Multimedia Appendix 1, the structure of the original data was preserved in different modes upon synthesis.

**Figure 4:**
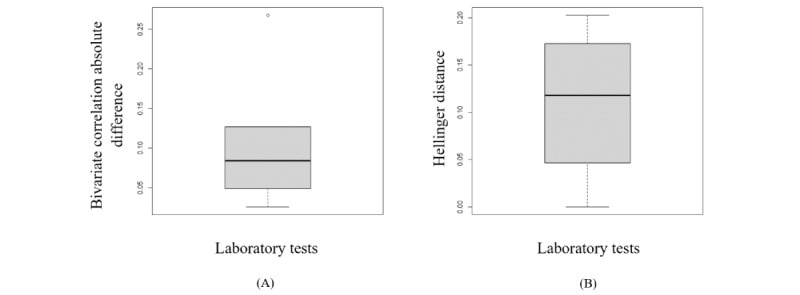
The box plots display the variation of the Hellinger distance and the Pearson correlation between the original variables and the synthetic variables generated by sequential decision trees. (A) This is the box plot of the absolute differences in bivariate correlations between the real and synthetic data. Smaller values indicate that the bivariate relationships in the data have been greatly preserved during the generation of synthetic data. (B) This is the box plot of the Hellinger distance between the original variables and the synthetic variables. This shows the similarity of the univariate distributions between the real and synthetic data. This is a value between 0 and 1, with lower values indicating similarity between the univariate distributions of the real and synthetic variables.

Figure 4 of the variation of the Hellinger distance also shows that synthetic variables from sequential decision trees are derived from a similar distribution as the original variables. However, the copula performed slightly better in capturing the correlations between the variables. The RMSDC computed for this experiment was 0.078.

The summary presented in Table 4 shows that the range of variables has significantly improved compared to the previous copula analysis, refer to Table 3 for the summary of the original data.

The following is the result of MCMC method using the HMC algorithm: if the distribution of the HMC model is well specified, the resulting outcome will be more effective.

**Figure 5:**
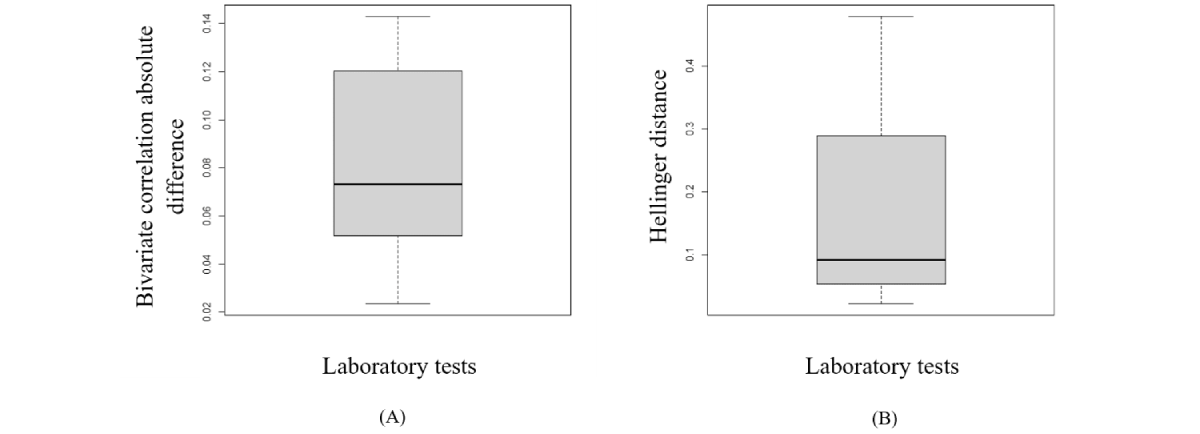
The box plots display the variation of the Hellinger distance and Pearson correlation between the original variables and the Hamiltonian Monte Carlo synthetic variables. (A) This is the box plot of the absolute differences in bivariate correlations between the real and synthetic data. Smaller values indicate that the bivariate relationships in the data have been greatly preserved during the generation of synthetic data. (B) This is the box plot of the Hellinger distance between the original variables and the synthetic variables. This shows the similarity of the univariate distributions between the real and synthetic data. This is a value between 0 and 1, with lower values indicating similarity between the univariate distributions of the real and synthetic variables.

Section S6 in Multimedia Appendix 1 indicates that the structure of the synthetic data in different modes is comparable to that of the original data. Furthermore, an RMSDC of 0.071 was obtained. We expected the HMC to perform well on the Gaussian latent space, which is defined by our Gaussian model. Defining a proper model distribution for the HMC would significantly enhance the findings. Figure 5 illustrates that HMC was slightly better at capturing the correlations between variables.

On the basis of Table 5, the summary of HMC synthetic variables is similar to that of the other methods. The maximum values of the variables dropped in the synthetic data compared to the actual data in Table 3.

**Figure 6:**
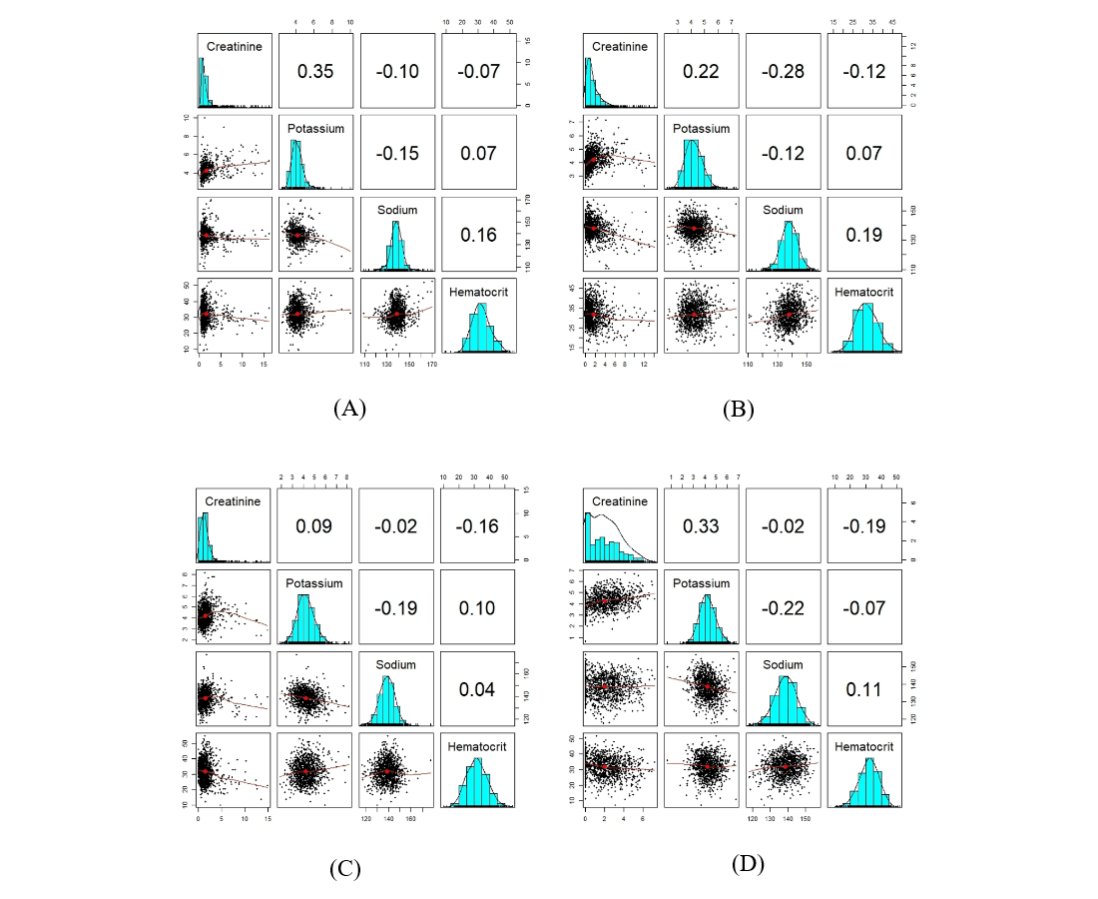
The plots show the correlation and distribution of the original and synthetic variables. A correlation matrix displays bivariate scatter plots of the adjacent variables below the diagonal, histograms of the data distribution of the respective variables on the diagonal, and the Pearson correlation above the diagonal. Ellipses specify the direction of the correlation. The information regarding the relationship between the 2 selected variables is always perpendicular to each other. (A) This is the plot of the original variables. (B) This is the plot of synthetic variables generated by copula. (C) This is the plot of synthetic variables generated by sequential decision trees. (D) This is the plot of synthetic variables generated by Hamiltonian Monte Carlo.

Finally, we present Figure 6 and section S7 in Multimedia Appendix 1 for an easier comparison of the 3 sampling techniques on GCP decomposition with Gaussian loss. In Figure 6, we can observe the dependency structure and univariate distribution of both synthetic and original variables simultaneously.

The findings and figures demonstrate that all 3 synthetic data sets have similar statistical properties in terms of dependency and univariate distributions. However, the copula and sequential trees performed slightly better than the MCMC technique when the HMC algorithm was used. In brief, we need to define a proper distribution that corresponds to the latent space to obtain decent results through HMC sampling. The Gaussian loss is the ideal loss function for simulating from the patient factor matrix using sequential trees, but the observed data must be standardized beforehand. Further analysis, which we have not included in this paper, has shown that it is preferable to use empirical CDF marginals instead of parametric ones when sampling the patient factor matrix by copula. The outcomes of generating synthetic data using β-loss in GCP decomposition can be found in section S3 in Multimedia Appendix 1.

In the following section, we will provide the results of generating synthetic data with an additional number of patients compared to the original data set.

**Table 2:** A summary of the copula’s synthetic variables.

Metric	Variables			
	Creatinine	Potassium	Sodium	Hematocrit
Minimum^a^	0	2.23	110.6	13.64
Median (IQR)	1.08 (0.52-2.21)	4.17 (3.77-4.62)	138 (134.5-141.9)	31.64 (27.97-35.75)
Mean (SD)	1.67 (1.58)	4.22 (0.59)	138 (6.2)	31.9 (4.1)
Maximum^b^	14.1	7.32	157.5	48.49

^a^Minimum: minimum of data.

^b^Maximum: maximum of data.

**Table 3:** A summary of the original variables.

Metric	Variables			
	Creatinine	Potassium	Sodium	Hematocrit
Minimum^a^	0.2	2.5	109	9.2
Median (IQR)	1 (0.7-1.6)	4.1 (3.8-4.51)	138 (135-141.6)	31.6 (28.1-35.7)
mean (SD)	1.64 (1.6)	4.23 (0.62)	138.4 (6.5)	32.08 (5.82)
Maximum^b^	16.2	10	170	52.6

^a^Minimum: minimum of data.

^b^Maximum: maximum of data.

**Table 4:** A summary of the sequential decision trees’ synthetic variables.

Metric	Variables			
	Creatinine	Potassium	Sodium	Hematocrit
Minimum^a^	0	1.88	116.4	10.09
Median (IQR)	1.25 (0.67-1.86)	4.18 (3.7-4.71)	138.8 (134.2-143.3)	31.68 (27.01-36.38)
Mean (SD)	1.53 (1.62)	4.23 (0.58)	138.7 (6.6)	31.87 (5.8)
Maximum^b^	14.84	8.16	177.4	54.8

^a^Minimum: minimum of data.

^b^Maximum: maximum of data.

**Table 5:** A summary of the Hamiltonian Monte Carlo’s synthetic variables.

Metric	Variables			
	Creatinine	Potassium	Sodium	Hematocrit
Minimum^a^	0	0.66	117.8	7.22
Median (IQR)	1.85 (0.6-3.1)	4.19 (3.74-4.7)	138.6 (134.5-142.8)	32.18 (27.85-36.47)
Mean (SD)	2.01 (1.4)	4.23 (0.54)	138.5 (5.02)	31.93 (4.8)
Maximum^b^	7.24	6.78	156.9	51.63

^a^Minimum: minimum of data.

^b^Maximum: maximum of data.

### Experiments on Continuous Data With a Different Number of Patients in Synthetic Data Compared to Original Data

We can generate different numbers of patients in the synthetic data using all 3 patient factor matrix simulations. However, we only applied the sequential trees approach, and the results are as follows: the findings indicate that the dependency and univariate structure of the original variables are well maintained in the synthetic data.

The following are the outcomes of generating 250 patients from the patient factor matrix using sequential trees: the patient factor matrix is derived from the GCP decomposition in the previous experiment. The original data set is dense, consisting of 226 patients, and is the same data set used in the previous experiment.

**Figure 7:**
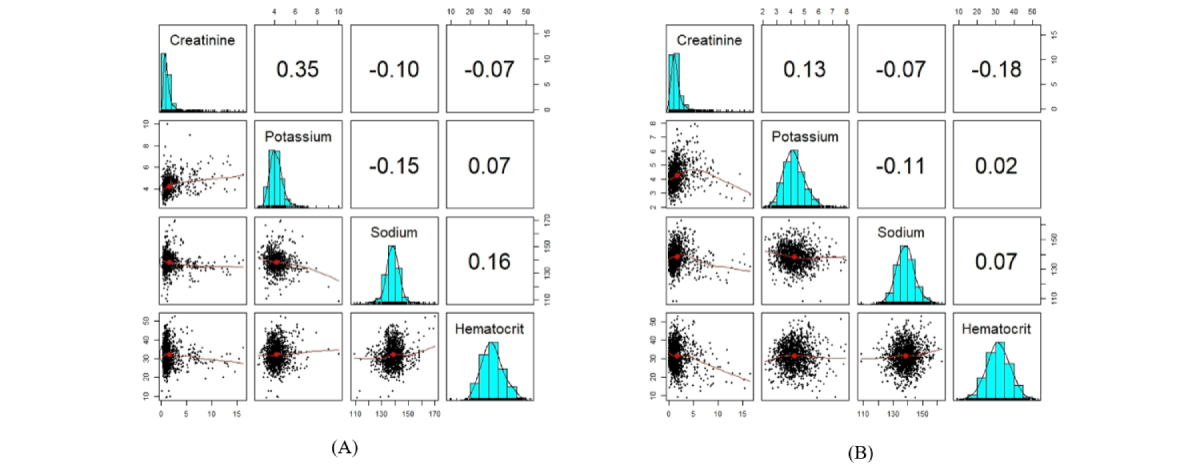
The plots show the correlation and distribution of sequential decision trees’ synthetic variables as well as the original variables. A correlation matrix displays bivariate scatter plots of the adjacent variables below the diagonal, histograms of the data distribution of the respective variables on the diagonal, and the Pearson correlation above the diagonal. Ellipses specify the direction of the correlation. The information regarding the relationship between the 2 selected variables is always perpendicular to each other. (A) This is the plot of the original variables. (B) This is the plot of the synthetic variables.

**Figure 8:**
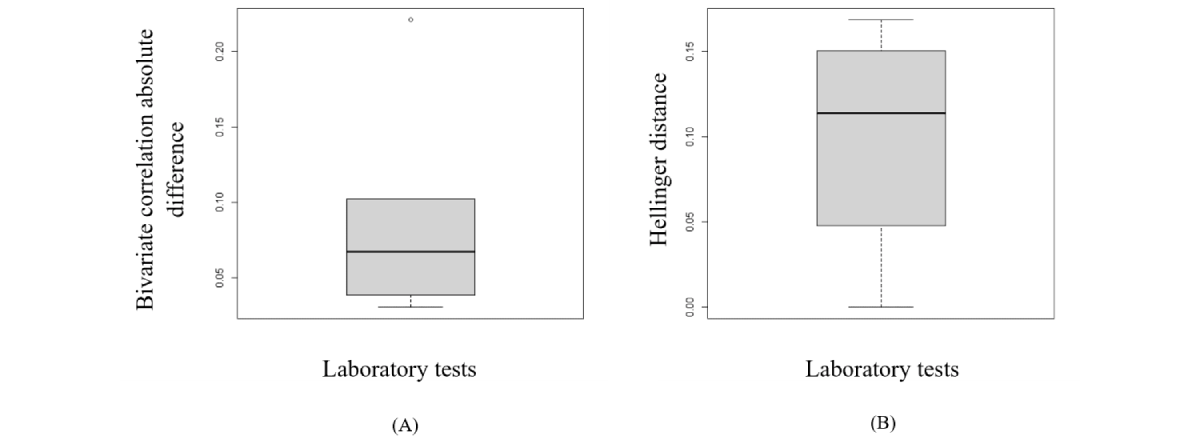
The box plots display the variation of the Hellinger distance and the Pearson correlation between the original variables and the sequential decision trees’ synthetic variables. (A) This is the box plot of the absolute differences in bivariate correlations between the real and synthetic data. Smaller values indicate that the bivariate relationships in the data have been greatly preserved during the generation of synthetic data. (B) This is the box plot of the Hellinger distance for all variables between the original and synthetic data sets. This shows the similarity of the univariate distributions between the real and synthetic data. This is a value between 0 and 1, with lower values indicating similarity between the univariate distributions of the real and synthetic variables.

Figure 7 indicates that the Pearson correlations and univariate distributions were similar between the synthetic and original data sets. Figure 8 illustrates that the synthetic variables created by sequential decision trees are derived from a distribution similar to that of the original variables. The structure of the generated data in different modes is included in section S8 in Multimedia Appendix 1, which shows similar results to those of the analyses presented here. The RMSDC computed for this experiment was 0.066.

In this scenario, the summary presented in Table 6 also demonstrates that the ranges of the synthetic variables are comparable to those of the original ones in Table 3.

On the basis of the results of this section, we are optimistic that our model may perform even better when generating larger data sets.

**Table 6:** A summary of sequential decision trees’ synthetic variables.

Metric	Variables			
	Creatinine	Potassium	Sodium	Hematocrit
Minimum^a^	0	2.16	108.3	8.97
Median (IQR)	1.22 (0.76-1.88)	4.18 (3.71-4.72)	138.4 (134.4-142.2)	31.44 (27.37-35.87)
Mean (SD)	1.67 (1.6)	4.26 (0.61)	138.4 (5.6)	31.56 (5.83)
Maximum^b^	16.42	7.94	163.1	53.04

^a^Minimum: minimum of data.

^b^Maximum: maximum of data.

### Experiments on Continuous Data With Missing Observations

The following is a trial on the continuous data derived from the MIMIC-III data set without imputation. As mentioned earlier, the data set consists of 226 patients, 4 laboratory tests, and 36 clinical visits, with 21% of the observations missing. We have previously described the approach for synthesizing this sort of data. In this experiment, we attempted to sample the patient factor matrix using sequential trees and generate the same sample size of 226 patients as in the original data. We performed GCP factorization with Gaussian loss and *R*=100. Then, we applied the CP via an alternating least square decomposition to the missing tensor with *R*=50, where the loss function was also Gaussian.

**Figure 9:**
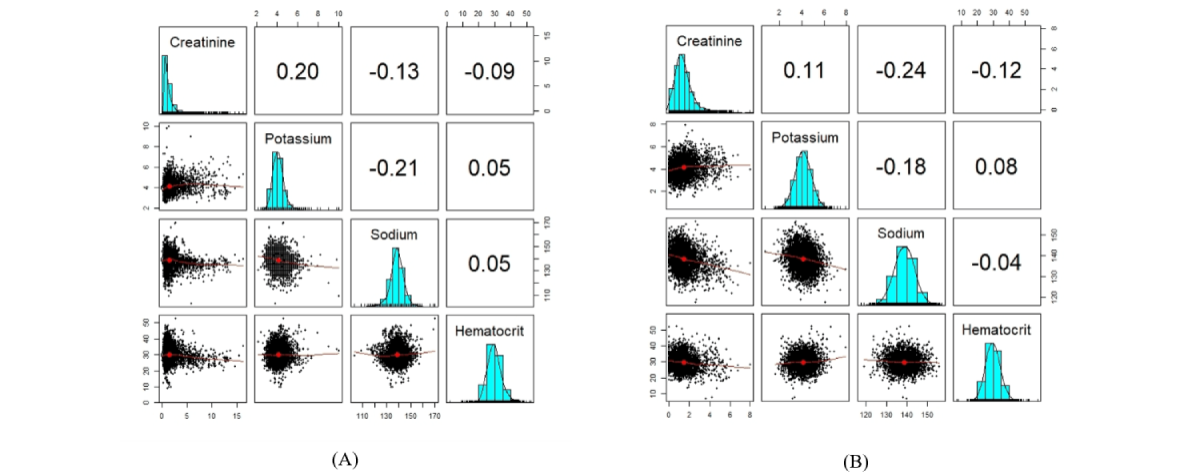
The plots show the correlation and distribution of sequential decision trees’ synthetic variables as well as the original variables. A correlation matrix displays bivariate scatter plots of the adjacent variables below the diagonal, histograms of the data distribution of the respective variables on the diagonal, and the Pearson correlation above the diagonal. Ellipses specify the direction of the correlation. The information regarding the relationship between the 2 selected variables is always perpendicular to each other. (A) This is the plot of the original variables. (B) This is the plot of the synthetic variables.

**Figure 10:**
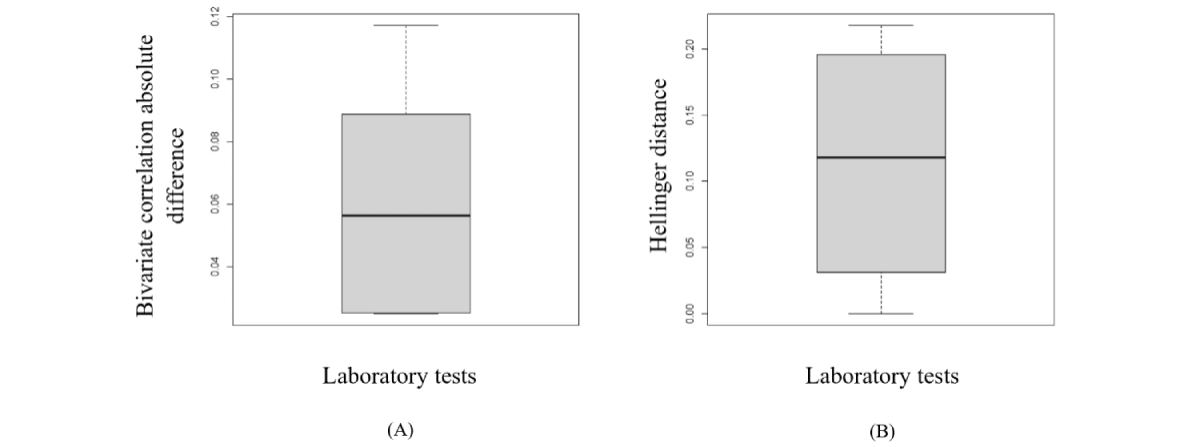
The box plots display the variation of the Hellinger distance and the Pearson correlation between the original variables and the sequential decision trees’ synthetic variables. (A) This is the box plot of the absolute differences in bivariate correlations between the real and synthetic data. Smaller values indicate that the bivariate relationships in the data have been greatly preserved during the generation of synthetic data. (B) This is the box plot of the Hellinger distance for all variables between the original and synthetic data sets. This shows the similarity of the univariate distributions between the real and synthetic data. This is a value between 0 and 1, with lower values indicating similarity between the univariate distributions of the real and synthetic variables.

The structure of the synthetic and original data sets in different modes is similar, as shown in section S9 in Multimedia Appendix 1. Furthermore, Figures 9 and 10 indicate that the dependency structure is preserved, and the marginal fitting is quite comparable in both the synthetic and original data sets. This demonstrates that our proposed model for EHR synthesis performs well.

According to the summary presented in Tables 7 and 8, the missing percentages in the synthetic and actual data are 46% and 21%, respectively. It appears to be almost double. As the missing tensor is binary, we can try to factorize it to determine if there are any possible improvements in the outcomes.

**Table 7:** A summary of the sequential decision trees’ synthetic variables.

Metric	Variables			
	Creatinine	Potassium	Sodium	Hematocrit
Minimum^a^	0	0.71	117.5	7.07
Median (IQR)	1.3 (0.85-1.87)	4.12 (3.64-4.62)	138.6 (135.3-142)	29.64 (26.58-32.77)
Mean (SD)	1.47 (0.97)	4.13 (0.76)	138.6 (5.02)	29.82 (4.8)
Maximum^b^	7.97	7.94	156.8	57.9
#NA’s^c^	3893	3636	3733	3661

^a^Minimum: minimum of data.

^b^Maximum: maximum of data.

^c^#NA’s: the number of missing values.

**Table 8:** A summary of the original variables.

Metric	Variables			
	Creatinine	Potassium	Sodium	Hematocrit
Minimum^a^	0	2.1	103	2
Median (IQR)	1 (0.6-1.6)	4 (3.7-4.4)	139 (135.8-142)	29.6 (26.9-32.5)
Mean (SD)	1.52 (1.67)	4.1 (0.58)	138.7 (5.67)	29.86 (4.62)
Maximum^b^	16.2	10	170	52.6
#NA’s^c^	2050	1532	1752	1515

^a^Minimum: minimum of data.

^b^Maximum: maximum of data.

^c^#NA’s: the number of missing values.

### Experiments on Continuous Data With Irregular Clinical Visits

To create irregularity in clinical visits, a subset of the continuous data was created by choosing the first 10 observations. Those outcomes are presented in this section, and we have previously elaborated on the method used for addressing this particular scenario. The GCP decomposition was performed with Gaussian loss and *R*=30, resulting in a mean square error of approximately 0.004. The experiment was conducted using the sequential decision trees approach to sample the patient factor matrix. The RMSDC was computed as 0.14.

**Figure 11:**
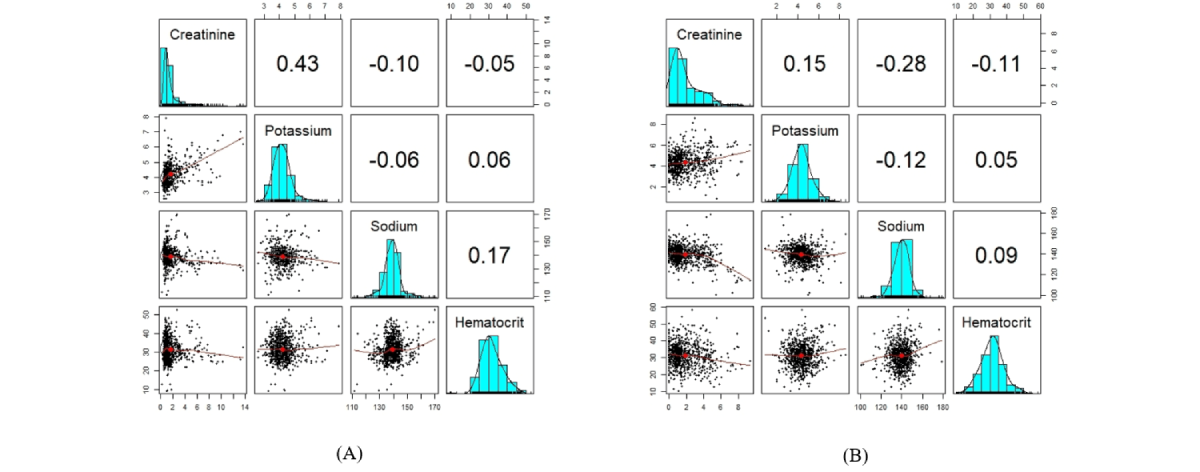
The plots show the correlation and distribution of variables generated by sequential trees and the original ones. A correlation matrix displays bivariate scatter plots of the adjacent variables below the diagonal, histograms of the data distribution of the respective variables on the diagonal, and the Kendall correlation above the diagonal. Ellipses specify the direction of the correlation. The information regarding the relationship between the 2 selected variables is always perpendicular to each other. (A) This is the plot of the original variables. (B) This is the plot of the synthetic variables.

**Figure 12:**
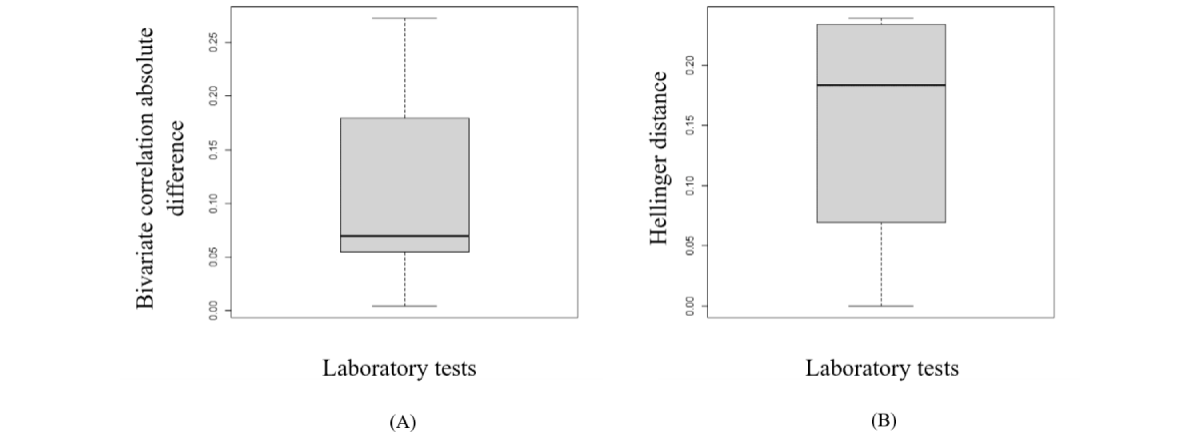
The box plots display the variation of the Hellinger distance and the Kendall correlation between the original variables and the sequential decision trees’ synthetic variables. (A) This is the box plot of the absolute differences in bivariate correlations between the real and synthetic data. Smaller values indicate that the bivariate relationships in the data have been greatly preserved during the generation of synthetic data. (B) This is the box plot of the Hellinger distance for all variables between the original and synthetic data sets. This shows the similarity of the univariate distributions between the real and synthetic data. This is a value between 0 and 1, with lower values indicating similarity between the univariate distributions of the real and synthetic variables.

Upon analyzing the results in Figures 11 and 12, we found that the process of generating synthetic data generally maintained bivariate relationships and univariate distributions in the data.

When analyzing Tables 9 and 10, it was found that the provided descriptive statistics have remained comparable throughout the process of generating synthetic data.

**Table 9:** A summary of the sequential decision trees’ synthetic variables.

Metric	Variables			
	Creatinine	Potassium	Sodium	Hematocrit
Minimum^a^	0	0.87	100.3	10.62
Median (IQR)	1.31 (0.76-2.82)	4.31 (3.73-4.88)	139.8 (134.9-144.6)	31.43 (27.02-35.21)
Mean (SD)	1.9 (1.18)	4.34 (0.56)	139.5 (5.11)	31.36 (5.7)
Maximum^b^	9.35	8.59	178.9	58.15

^a^Minimum: minimum of data.

^b^Maximum: maximum of data.

**Table 10:** A summary of the original variables.

Metric	Variables			
	Creatinine	Potassium	Sodium	Hematocrit
Minimum^a^	0.2	2.6	111.2	9.2
Median (IQR)	1.07 (0.76-1.7)	4.15 (3.8-4.5)	139 (135.6-142)	31 (27.83-35.1)
Mean (SD)	1.6 (1.64)	4.21 (0.62)	139 (6.53)	31.61 (5.82)
Maximum^b^	13.6	7.9	170	52.6

^a^Minimum: minimum of data.

^b^Maximum: maximum of data.

### Experiments on Categorical Dense Data

The categorical data contain 2 variables: “admission type” and “admission location.” The GCP decomposition was implemented using 2 different loss functions: the Poisson log link, the results of which we discuss in section S10 in Multimedia Appendix 1, and the Gaussian loss function. Initially, a series of postprocessing steps were conducted. The outcomes of the synthesis, which apply the Gaussian loss function and use HMC, are presented in the subsequent section. In this experiment, *R*=10 was obtained.

**Figure 13:**
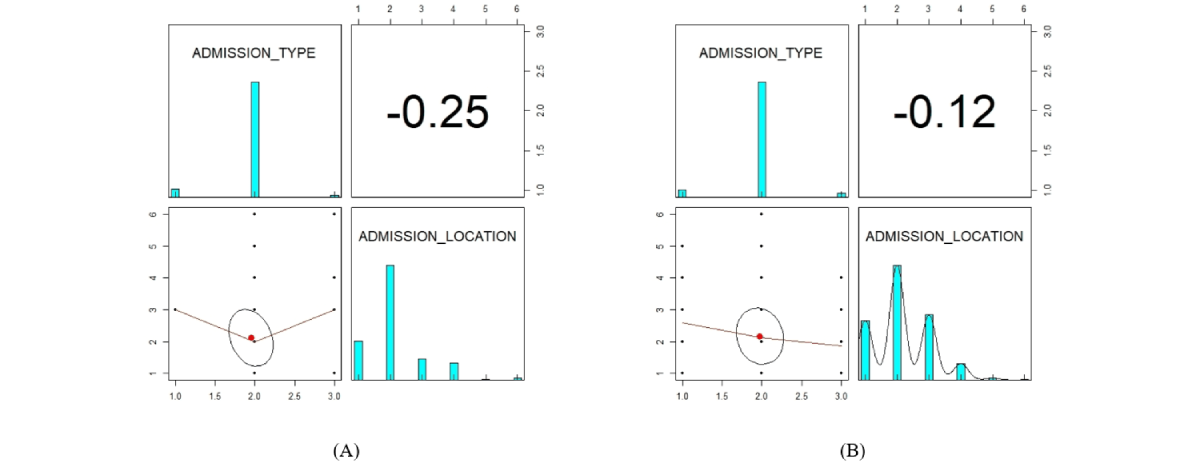
The plots show the Kendall correlation and distribution of the Hamiltonian Monte Carlo’s synthetic variables as well as the original variables. A correlation matrix displays bivariate scatter plots of the adjacent variables below the diagonal, a bar chart of the data distribution of the respective variables on the diagonal, and the Kendall correlation above the diagonal. Ellipses specify the direction of the correlation. (A) This is the plot of the original variables. (B) This is the plot of the synthetic variables.

**Figure 14:**
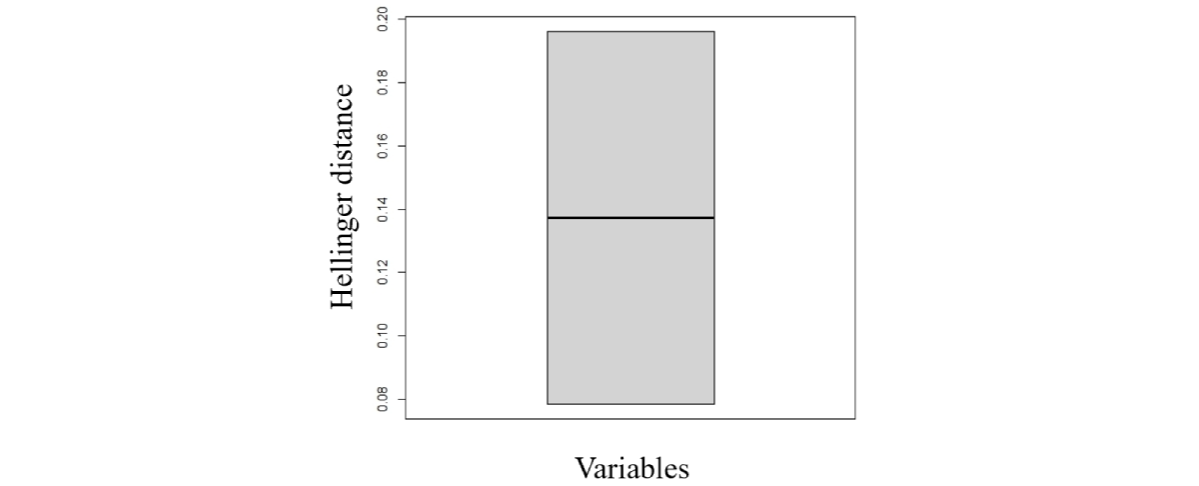
The box plot shows the variation of Hellinger distance for all variables between the original and the Hamiltonian Monte Carlo synthetic data sets. This shows the similarity of the univariate distributions between the real and synthetic data. This is a value between 0 and 1, with lower values indicating similarity between the univariate distributions of the real and synthetic variables.

The structure of the generated data in different modes is included in section S11 in Multimedia Appendix 1. The Hellinger distance and Kendall correlation were calculated for the categorical variables as shown in Figures 13 and 14. All the findings demonstrate that the generative model is applicable to any sort of variable.

## Discussion

### Summary

Our objective was to develop and validate a generative model that produces synthetic longitudinal health data. We constructed a model by using a GCP tensor decomposition and sampling from its latent factor matrix, which contains factors related to patients.

We applied the GCP decomposition because tensor decompositions offer interpretability and flexibility in handling high-dimensional data, including massive and heterogeneous EHR data sets. However, the most sensible and acceptable privacy concepts were undermined because of the one-to-one mapping and direct correspondence between the entries of the GCP model and the entries of the original data. Thus, by simulating and modeling the latent factor matrix of GCP decomposition associated with patients, we could address privacy concerns.

We proposed 3 methods for synthesizing and simulating the patient’s factor matrix: sequential trees, Gaussian copula, and HMC. These techniques appear to be the best options for data synthesis and simulation, particularly when working with complex and small data sets, such as the patient factor matrix in our model.

The model was validated through several experiments conducted on various data structures. We assessed the similarity between our synthetic data and the real data by conducting utility assessments. The assessments involved evaluating the structure and general patterns present in the data, such as the dependency structure, the descriptive statistics, and the marginal distributions.

### Limitations

In this study, we were not able to use the huge data set. In addition, we could not investigate further simulation and sampling techniques for the patient factor matrix due to time constraints. We focused on longitudinal health data in our model. However, there are also other types of longitudinal data, such as transactions in financial data sets, that occur over time.

Therefore, a future study could use a huge data set for this model and explore other techniques for synthesizing the patient factor matrix, such as GANs and recurrent neural network models. Another possible future work could involve conducting a more rigorous comparison between the original and synthetic data sets to evaluate both the generative model and the superior sampling approach. We could also look at the HMC sampling approach and see if we can improve its results by defining more appropriate distributions. We believe that our model could perform well on various types of longitudinal data sets. It would be interesting and valuable to carry out a future study to assess the effectiveness and feasibility of this model on different types of longitudinal data, such as financial data, as the current model has been developed and validated using health data.

### Conclusions

There is an increasing demand to access EHRs for secondary analysis. Data synthesis is one method that can address this demand and satisfy privacy concerns simultaneously. The objective of this study was to develop and validate a generative model for producing synthetic longitudinal health data. This was achieved using GCP tensor decomposition and sampling its latent factor matrix, which contains patient factors. All the simulation methods used in the generative model provided the same high level of performance in certain experiments. However, the sequential decision trees performed better when data standardization was used, and the Gaussian loss was used in the generalized CP decomposition. When applied to a non-Gaussian latent space, the copula was preferred. Our approach could also solve the problem of sampling patients from EHRs. This means that we could simulate different numbers of patients in the synthetic data set as well. On the basis of our findings, we highly recommend the standardization and decomposition of EHRs using Gaussian loss. This will ensure that the synthetic data is a true reflection of the original data set.

We successfully addressed the challenge of synthesizing massive longitudinal health data by synthesizing a significantly smaller nonlongitudinal data set instead. Thus, it is encouraging that our generative model could be applied to produce valuable synthetic data in various fields and areas of research.

Tensor decompositions have drawn growing attention because of their interpretability and flexibility in high-dimensional and heterogeneous data sets. In addition, they can easily be privatized. The GCP decomposition is the most popular tensor decomposition technique, which is ideal for large-scale and heterogeneous data sets. It has various applications beyond health data analysis, such as in predicting financial markets. There are significant benefits to banks and financial institutions when it comes to generating and using synthetic data. For instance, they gain the ability to analyze and test data without any cybersecurity or privacy concerns, which is crucial and saves a tremendous amount of time.

Therefore, we believe that our model can efficiently apply to various types of longitudinal data, including generating synthetic longitudinal financial data, such as synthetic transactions.

## Appendix

Multimedia Appendix 1 Detailed analysis results, loss functions, and algorithm descriptions.

## Abbreviations

CDF: cumulative distribution function
CP: canonical polyadic
EHR: electronic health record
GAN: generative adversarial network
GCP: generalized canonical polyadic
HMC: Hamiltonian Monte Carlo
MCMC: Markov chain Monte Carlo
RMSDC: root mean square difference between the actual and synthetic correlations
