# Supplementary material for: Creating High-Quality Synthetic Health Data: Framework for Model Development and Validation
Source: JMIR Form Res. 2024 Apr 22;8:e53241. doi: 10.2196/53241 (PMC11034549; doi:10.2196/53241)
Supplement: Multimedia Appendix 1 [file formative_v8i1e53241_app1.pdf]

## Section S1: The Choice of the Loss Functions in the GCP Tensor Decomposition

The choice of the loss function in the GCP tensor decomposition depends on how the original data is generated, which can be found below [1].

| Distribution | Data type | Link function | Loss function | Constraints |
|---|---|---|---|---|
| $\mathcal{N}(\mu, \sigma)$ | Continuous | $\ell(\mu) = \mu = m$ | $(x - m)^2$ | $x, m \in \mathbb{R}$ |
| Gamma$(k, \sigma)$ | Positive continuous | $\ell(\sigma) = k\sigma = m$ | $x/(m + \epsilon) + \log(m + \epsilon)$ | $x > 0, m \geq 0$ |
| Rayleigh $(\theta)$ | Nonnegative continuous | $\ell(\theta) = \sqrt{\pi/2}\theta = m$ | $2\log(m + \epsilon) + (\pi/4)(x/(m + \epsilon))^2$ | $x > 0, m \geq 0$ |
| Poisson $(\lambda)$ | Count | $\ell(\lambda) = \lambda = m$ | $m - x\log(m + \epsilon)$ | $x \in \mathbb{N}, m \geq 0$ |
| Bernoulli $(\rho)$ | Boolean | $\ell(\rho) = \rho/(1 - \rho) = m$ | $\log(m + 1) - x\log(m + \epsilon)$ | $x \in \mathbb{N}, m \in \mathbb{R}$ |
| NegBinom $(r, \rho)$ | Count | $\ell(\rho) = \rho/(1 - \rho) = m$ | $(r + x)\log(1 + m) - x\log(m + \epsilon)$ | $x \in \{0, 1\}, m \in \mathbb{R}$ |

Table 1: Loss functions

## Section S2: The Model Block of STAN

Here is the model block of STAN for sampling from the patient factor matrix using HMC. The $x$ and $x\_sim$ in the model block of STAN represent the patient factor matrix variables $\mathbf{a}_i$ and their simulation $\hat{\mathbf{a}}_i$, respectively, and $N$ is the number of patients.

```
data {
        int<lower=0> N;
        vector[R] x[N];
}

transformed data {
        vector[R] mu = rep_vector(0, R);
}

parameters {
        cholesky_factor_corr[R] chol;
        vector<lower=0>[R] sigma;
}

transformed parameters {
        matrix[R, R] chol_cov = diag_pre_multiply(sigma, chol);
}

model {
        chol ~ lkj_corr_cholesky(1);
        sigma ~ cauchy(0, 5);
        x ~ multi_normal_cholesky(mu, chol_cov);
}

generated quantities {
        vector[R] x_sim[N];
        for(i in 1:N) {
                x_sim[i] = multi_normal_cholesky_rng(mu, chol_cov);
        }
}
```

Textbox 1: The STAN model used in HMC sampling.

## Section S3: The Outcomes of Generating Synthetic Continuous Data Using $\beta$-loss in GCP Decomposition

In the following, we present the outcomes of synthetic continuous data generated by GCP using $\beta$-divergence with $\beta = 0.75$, $R = 15$, where the fit score and MSE were about .977 and 2.5, respectively. The dataset used in this experiment consists of 226 patients, 4 laboratory tests, and 36 clinical visits. It is the imputed version of the continuous dataset derived from the MIMIC dataset. As the MSE is not too small so it was expected that the result would not be outstanding, However, Copula and sequential trees performed better than HMC. As can be observed, all three recommended methods of patient factor matrix sampling have a much greater correlation than the real one. Copula, sequential trees, and the HMC results can be found in the following, respectively.

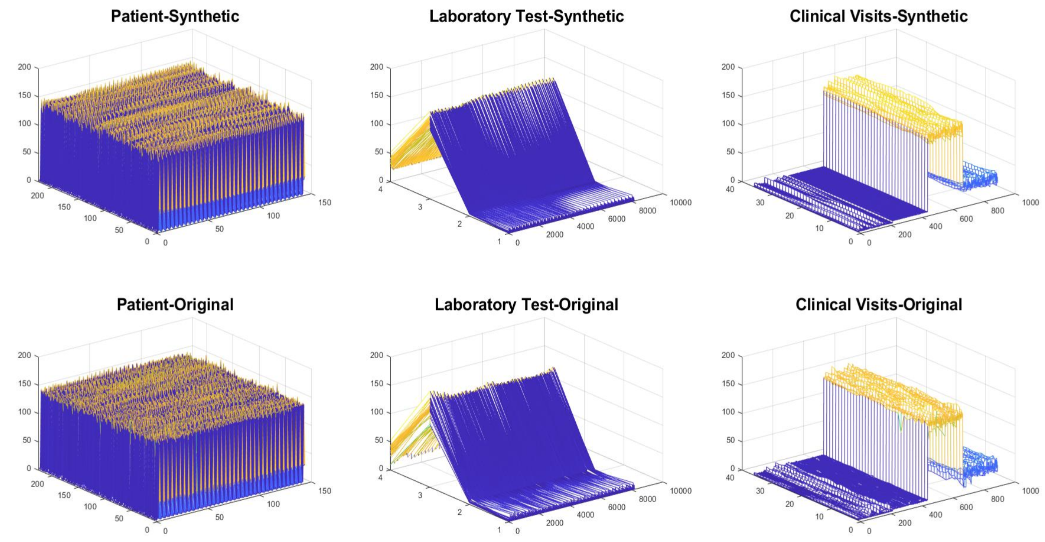


Figure 1: The different modes (Patients, Laboratory tests, and Clinical visits) of Copula's generated data and the original data are shown.

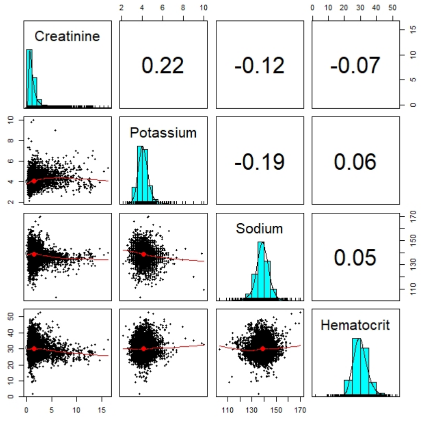


(a) Original variables

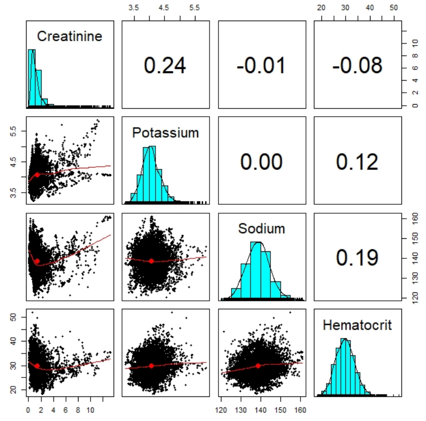


(b) Synthetic variables

Figure 2: The plots display the correlation and distribution of all variables in the generated data by Copula and the original dataset.

According to Figures 2 and 3, the dependency structure and distribution of the original variables are almost preserved in the synthetic data generated by Copula using empirical CDF marginals. The box plots for the variation of Hellinger distance and Pearson correlation between variables in the synthetic and original data in Figure 3 also represent the same. RMSDC=0.104 was obtained.

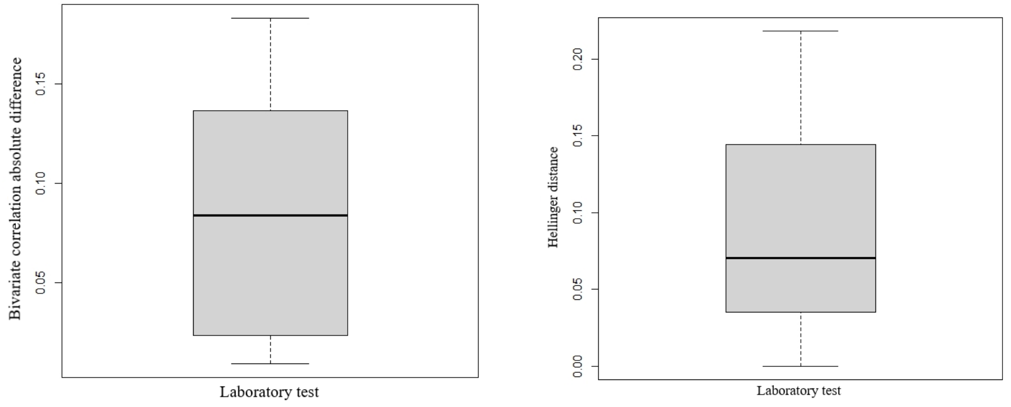


(a) Bivariate correlation absolute difference boxplot

(b) Hellinger distance boxplot

Figure 3: The variation of Hellinger distance and Pearson correlation between the original and Copula's synthetic variables.

According to the summary table below, the minimum of variables “Sodium” and “Hematocrit” are somewhat higher than the original.

| Metric | Variables | | | |
|---|---|---|---|---|
| | Creatinine | Potassium | Sodium | Hematocrit |
| Min | 0.09 | 3.21 | 120.2 | 18.33 |
| 1st Q | 0.64 | 3.86 | 134.5 | 26.99 |
| Median | 0.98 | 4.04 | 138.6 | 29.71 |
| Mean | 1.37 | 4.07 | 138.6 | 29.83 |
| 3rd Q | 1.52 | 4.25 | 142.5 | 32.42 |
| Max | 13.15 | 5.87 | 161.2 | 52 |

Table 2: The Copula's synthetic data summary.

| Metric | Variables | | | |
|---|---|---|---|---|
| | Creatinine | Potassium | Sodium | Hematocrit |
| Min | 0 | 2.1 | 103 | 2 |
| 1st Q | 0.62 | 3.74 | 135.4 | 27 |
| Median | 0.99 | 4.03 | 139 | 29.7 |
| Mean | 1.47 | 4.09 | 138.6 | 30.05 |
| 3rd Q | 1.6 | 4.4 | 142 | 32.7 |
| Max | 16.2 | 10 | 170 | 52.6 |

Table 3: The original data summary.

Here are the outcomes of sampling patient factor matrix of the previously mentioned GCP decomposition using sequential trees approaches.

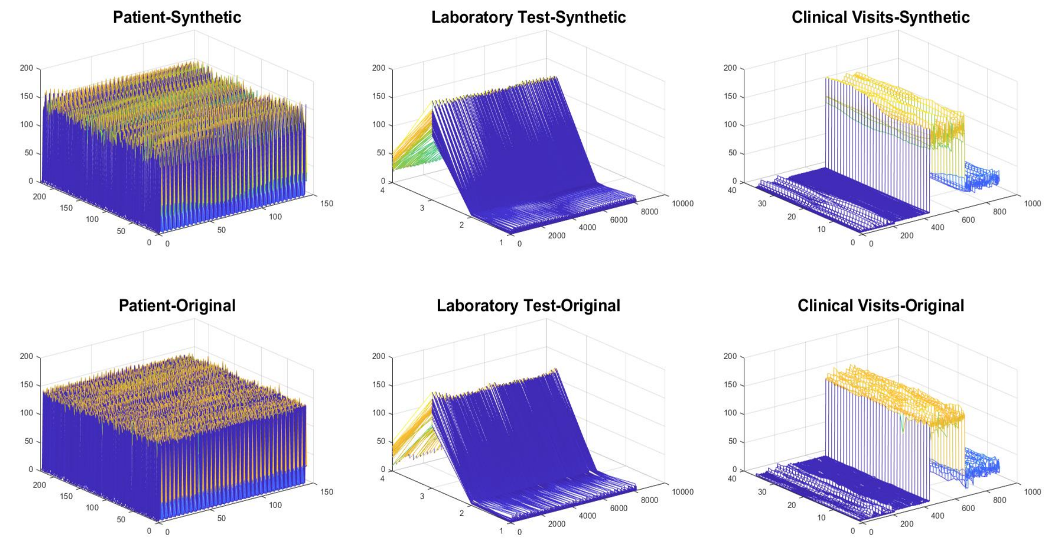


Figure 4: The different modes (Patients, Laboratory tests, and Clinical visits) of the sequential trees's generated data are shown.

The Figure 5 indicates that the univariate distributions are similar for each variables in synthetic and original datasets. The Figure 6 of the variation of Hellinger distance also shows that sequential decision trees' synthetic variables are derived from the similar distribution as the original variables. However, the Copula performed better in capturing the correlations between variables. The computed RMSDC for this experiment was 0.237.

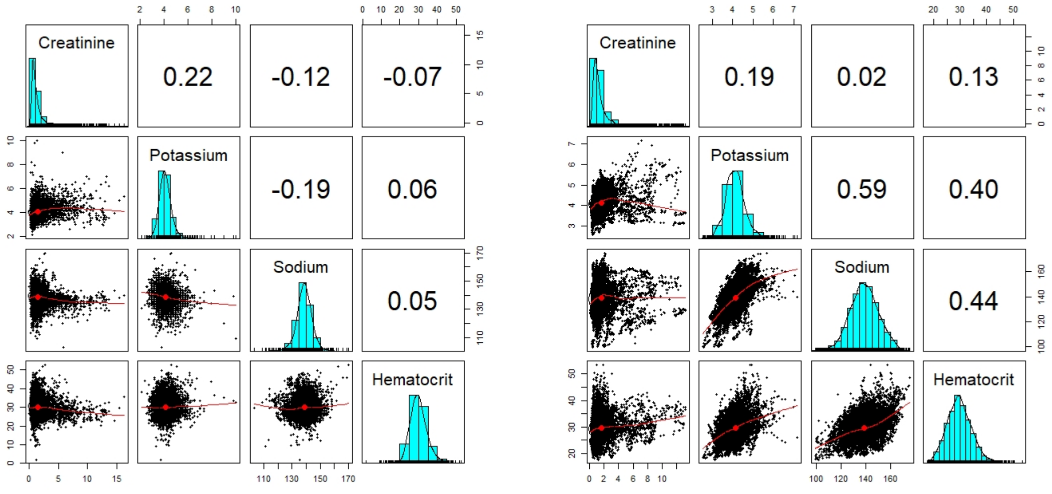


(a) Original variables

(b) Synthetic variables

Figure 5: The plot shows the correlation and distribution of the original data and the data generated by sequential decision trees.

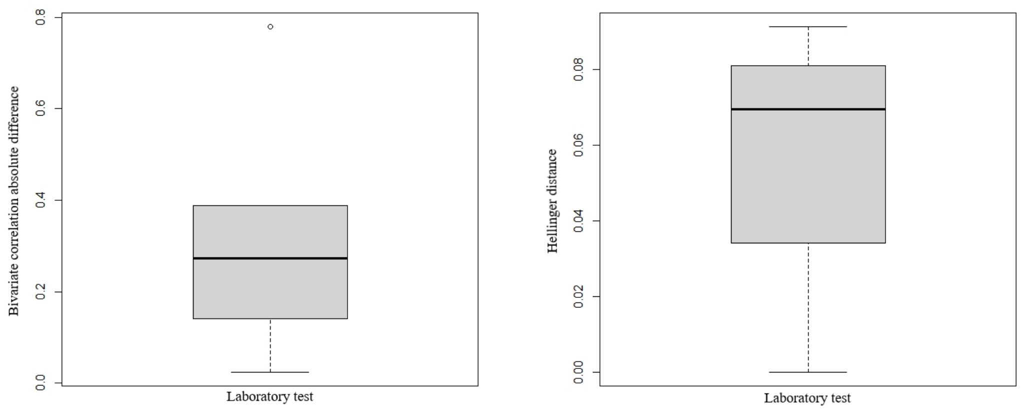


(a) Bivariate correlation absolute difference boxplot

(b) Hellinger distance boxplot

Figure 6: The variation of Hellinger distance and Pearson correlation between the original and sequential decision trees' synthetic variables.

The below summary displays that the range of variable "Sodium" has been significantly improved respect to the previous analysis using Copula, refer to Table 3 for the summary of the original dataset.

| Metric | Variables | | | |
|---|---|---|---|---|
| | Creatinine | Potassium | Sodium | Hematocrit |
| Min | 0.15 | 2.54 | 99.37 | 17.71 |
| 1st Q | 0.74 | 3.8 | 131.21 | 26.5 |
| Median | 1.1 | 4.1 | 138.75 | 29.57 |
| Mean | 1.65 | 4.13 | 138.82 | 29.79 |
| 3rd Q | 1.77 | 4.4 | 146.33 | 32.73 |
| Max | 13.15 | 7.18 | 175.29 | 53.31 |

Table 4: The sequential decision trees' synthetic data summary.

Here are the results of the Hamiltonian Monte Carlo performance on the dataset.If the distribution of the HMC model is properly defined, the outcome would be quite satisfactory.

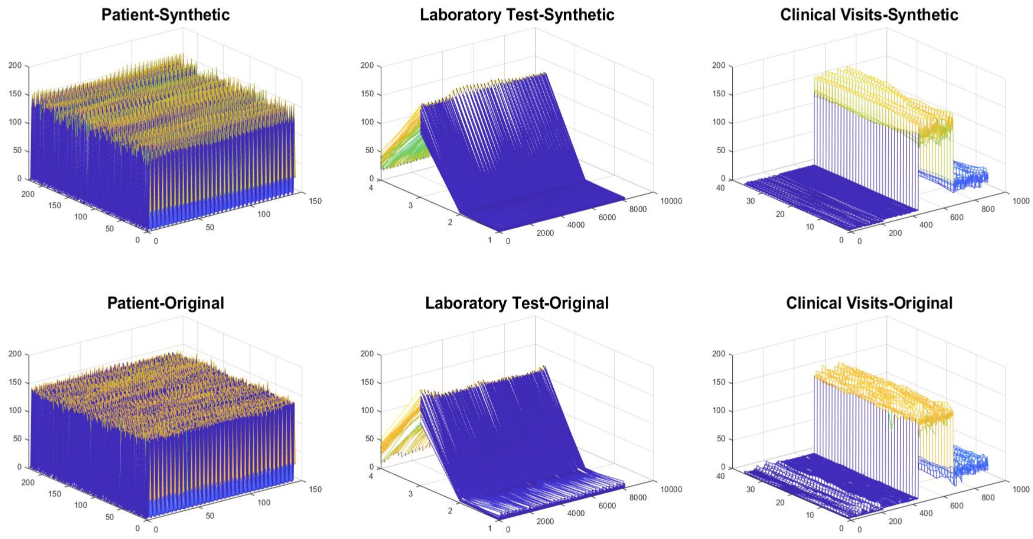


Figure 7: The different modes (Patients, Laboratory tests, and Clinical visits) of HMC's generated data are shown.

We did not expect HMC performing well here since the $\beta$-divergence loss causes a non-Gaussian latent space, and we won't get a good result even when standardizing the latent space. On the other hand, defining a proper model distribution for the HMC would considerably enhance the findings. However, due to the time constraints of this study, we were unable to test alternative model distribution such as Tweedie.

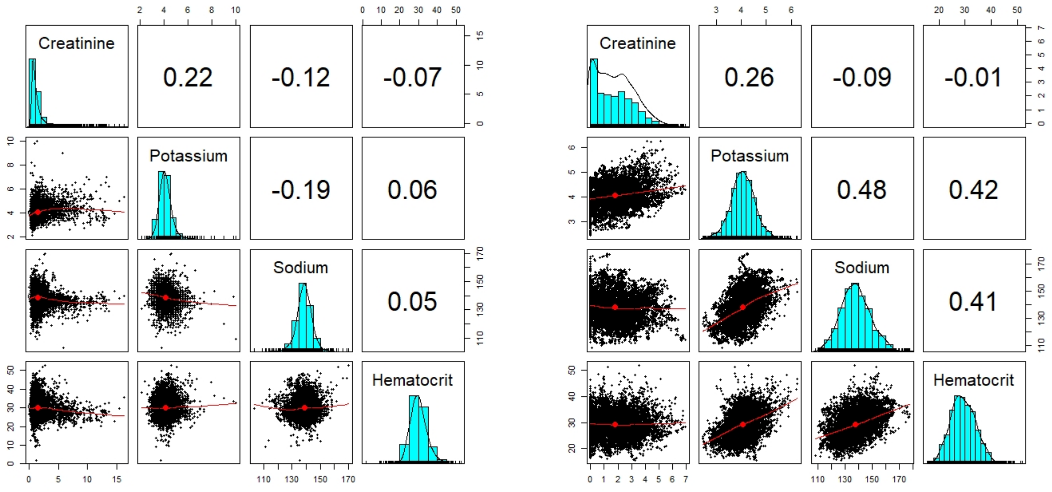


(a) Original variables

(b) Synthetic variables

Figure 8: The plot shows the correlation and distribution of the original data and the data generated by HMC.

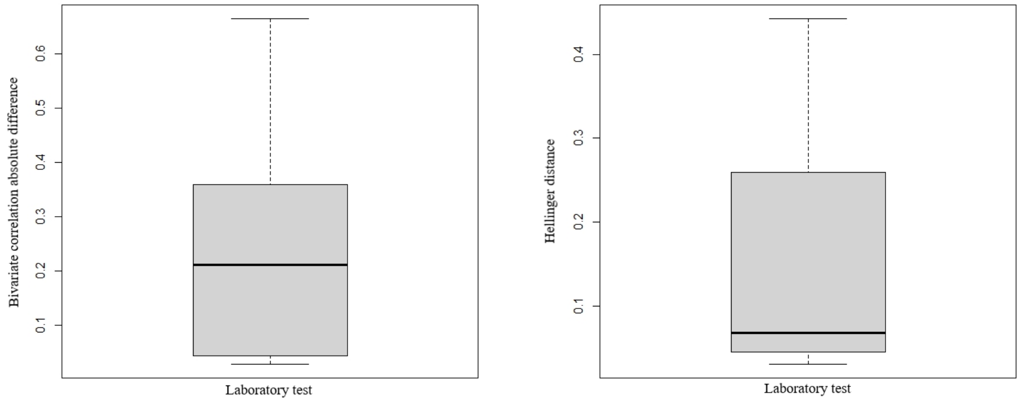


(a) Bivariate correlation absolute difference boxplot

(b) Hellinger distance boxplot

Figure 9: The variation of Hellinger distance and Pearson correlation between the original and HMC's synthetic variables.

The comparison of the summary below with Table 3 reveals that HMC performed poorly in this particular scenario.

| Metric | Variables | | | |
|---|---|---|---|---|
| | Creatinine | Potassium | Sodium | Hematocrit |
| Min | 0 | 2.54 | 108.3 | 15.48 |
| 1st Q | 0.58 | 3.75 | 130.6 | 25.91 |
| Median | 1.65 | 4.06 | 137.5 | 29.04 |
| Mean | 1.79 | 4.06 | 138 | 29.3 |
| 3rd Q | 2.78 | 4.37 | 145 | 32.62 |
| Max | 6.9 | 6.25 | 177.6 | 51.91 |

Table 5: The HMC's synthetic data summary.

At last, we provide Figure 10 to make it easier comparing the three sampling techniques on the GCP decomposition with $\beta$-divergence loss.

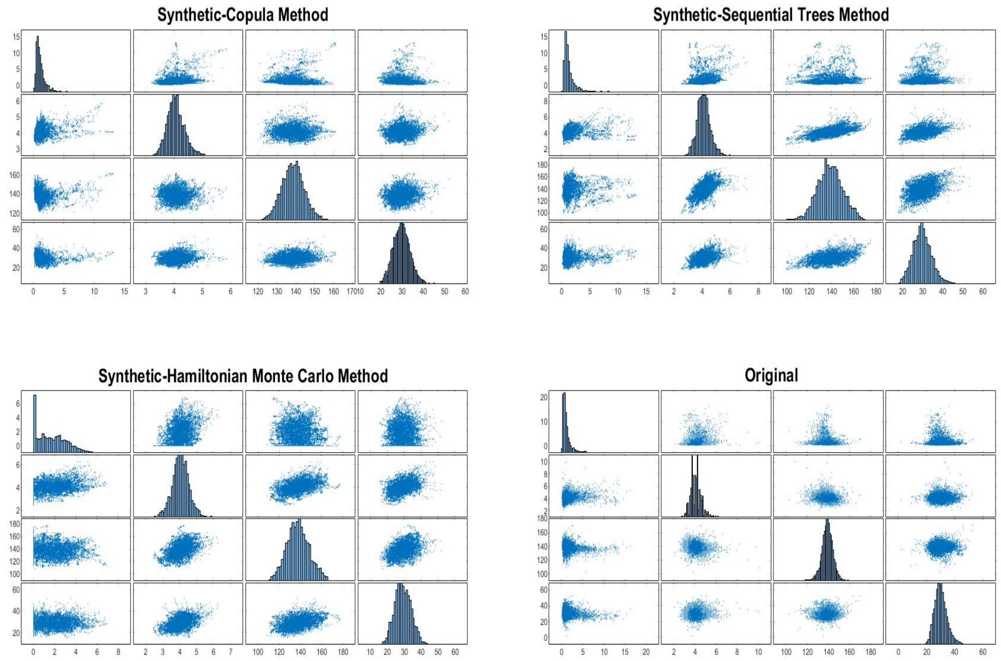


Figure 10: The distribution and scatter plots of original dataset and synthetic data generated using Copula, the sequential trees, and HMC.

## Section S4: Results Plot

The following plot displays the different tensor modes, illustrating how the synthetic data generated from the Copula with empirical CDF marginals almost preserves the structure and distribution of the original variables in trials on continuous dense data.

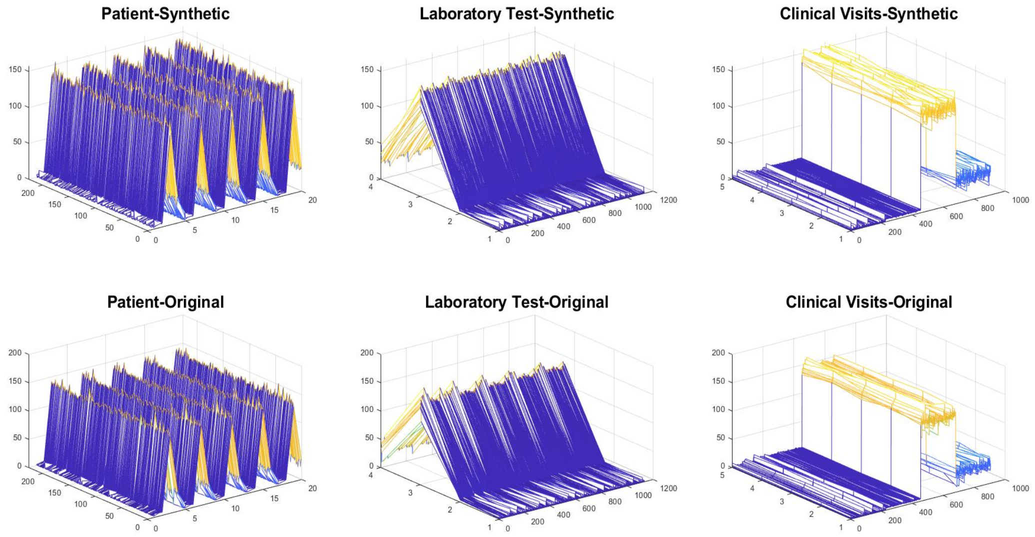


Figure 11: The different modes (Patients, Laboratory tests, and Clinical visits) of Copula's generated data and the original data.

The figure shows that the synthetic data generated from the Copula with empirical CDF marginals effectively maintains the distribution of the original variables in trials on continuous dense data, and the structure of the original data in different modes was preserved upon synthesis.

## Section S5: Results Plot

Here is an outcome of sampling patient factor matrix using sequential trees approach in experiments on continuous dense data.

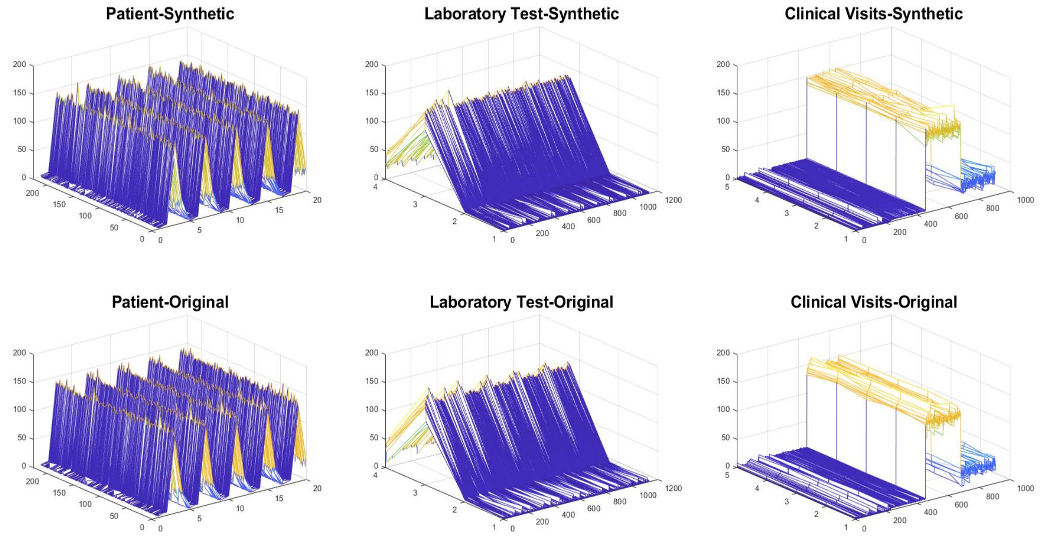


Figure 12: The different modes (Patients, Laboratory tests, and Clinical visits) of the sequential trees' generated data and the original data.

According to the above figure, the structure of the original data in different modes was preserved upon synthesis.

## Section S6: Results Plot

The following is a result of MCMC method using the Hamiltonian Monte Carlo in experiments on continuous dense data.

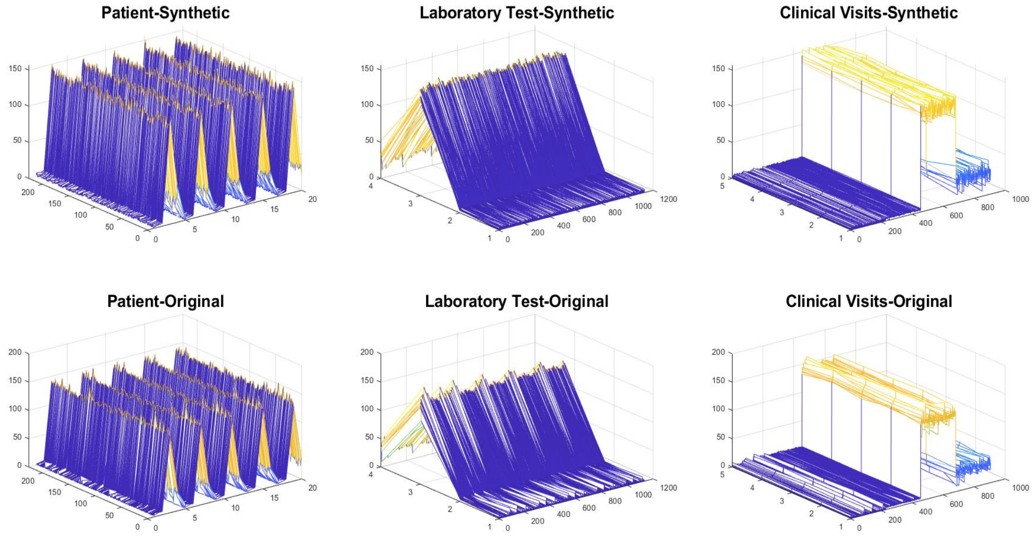


Figure 13: The different modes (Patients, Laboratory tests, and Clinical visits) of HMC's generated data and the original data.

The above figure indicates that the structure of the synthetic data in different modes is comparable to the original one.

## Section S7: Results Plot

We provide the below Figure to make it easier comparing the three sampling techniques on the GCP decomposition in experiments on continuous dense data.

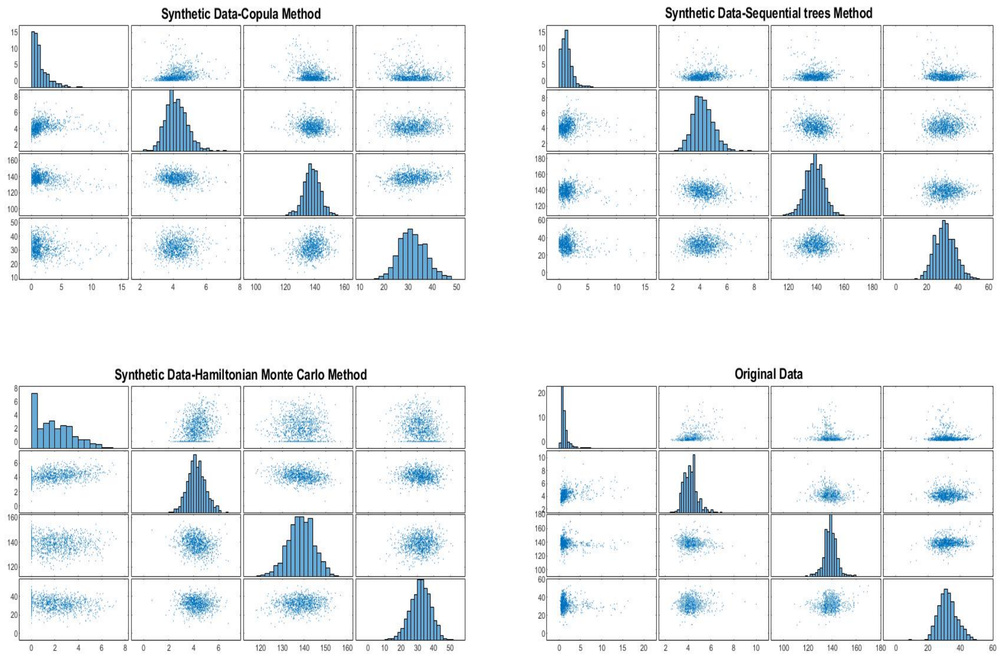


Figure 14: The distribution and scatter plots of the original variables and synthetic variables generated using Copula, sequential trees, and HMC in experiments on continuous dense data.

The figure demonstrates that all three synthetic datasets have similar statistical properties in terms of dependency and univariate distributions.

## Section S8: Results Plot

Here is an outcome of employing sequential trees to sample 250 patients from the patient factor matrix of the GCP decomposition, given that the original dataset includes 226 patients.

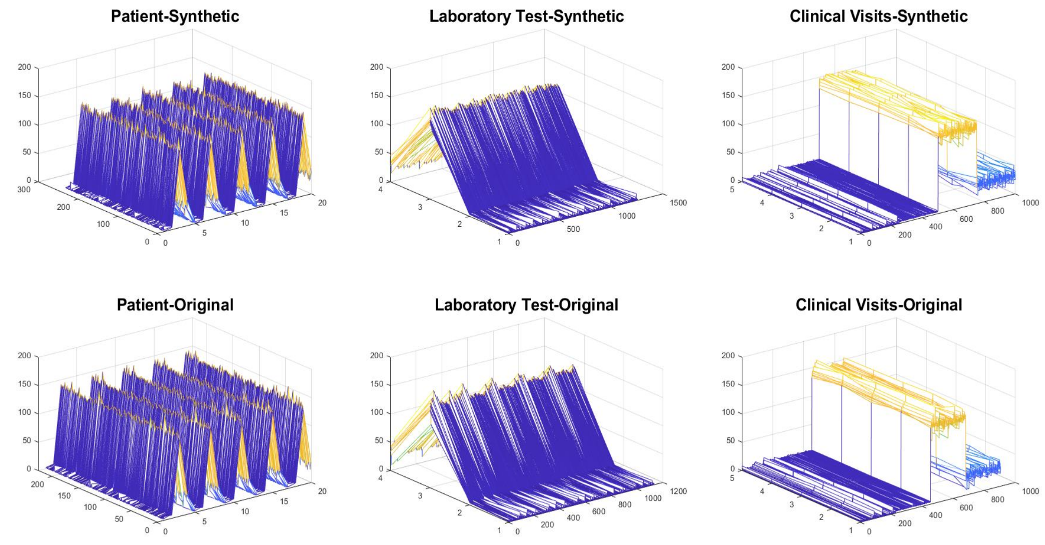


Figure 15: The different modes (Patients, Laboratory tests, and Clinical visits) of sequential trees' generated data and the original data.

The preceding figure shows that the structure of the synthetic data in different modes is comparable to the original one.

## Section S9: Results Plot

The following is a figure that resulted from sampling 226 patients in the patient factor matrix of the GCP decomposition using sequential trees. The original dataset has 21% missing observations.

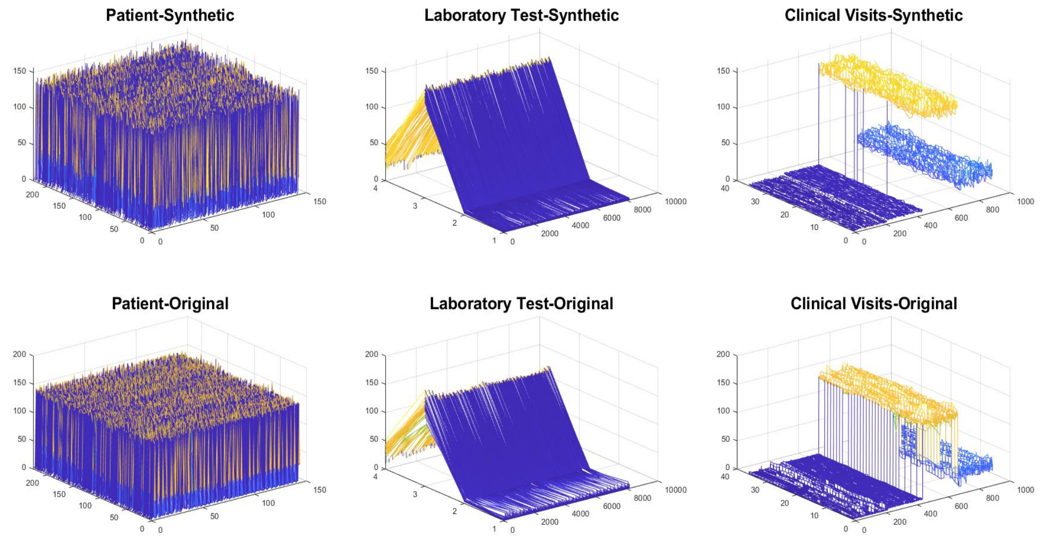


Figure 16: The different modes (Patients, Laboratory tests, and Clinical visits) of the original and sequential decision trees' synthetic data.

The structure of synthetic and original datasets in different modes are similar as shown here.

## Section S10: The Outcomes of Generating Synthetic Categorical Data Using Poisson log link in GCP Decomposition

Here are the results from generating categorical data using the GCP decomposition with a Poisson log link. In addition, the simulation for the patient factor matrix was conducted using HMC.

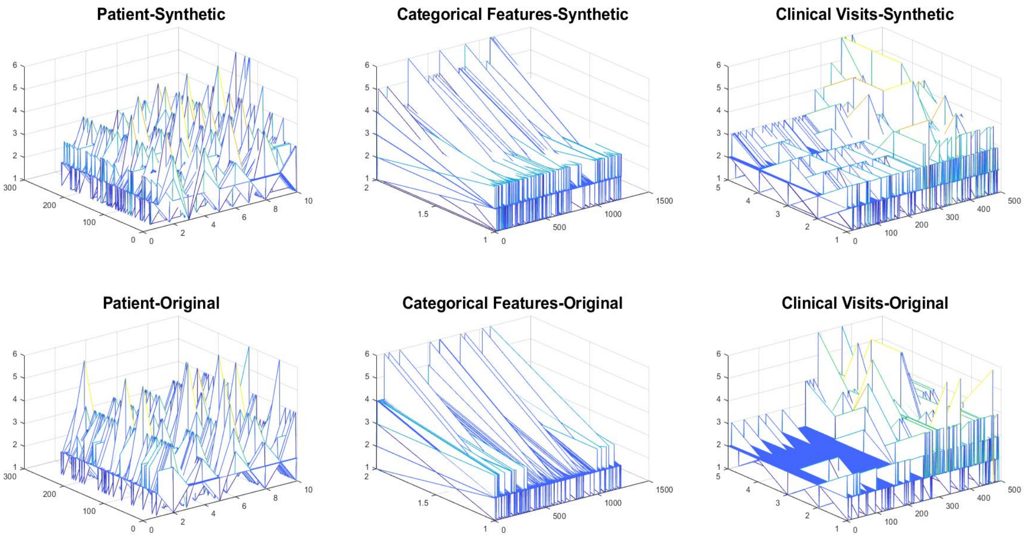


Figure 17: The different modes (Patients, Categorical features, and Clinical visits) of HMC's generated categorical data are shown.

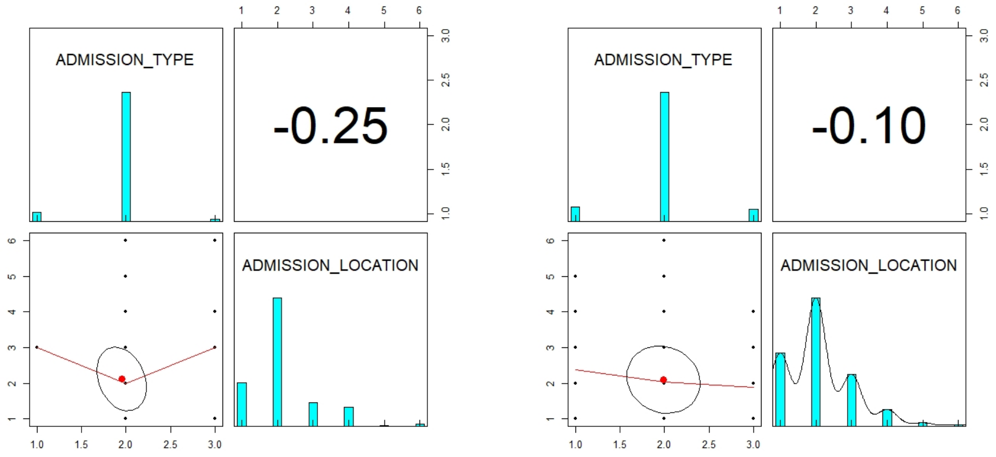


(a) Original variables

(b) Synthetic variables

Figure 18: The correlation and distribution plot illustration of HMC's generated data.

The Hellinger distance and Kendall correlation were computed for the categorical variables. All the results indicate that the generative model can be applied to any type of variable.

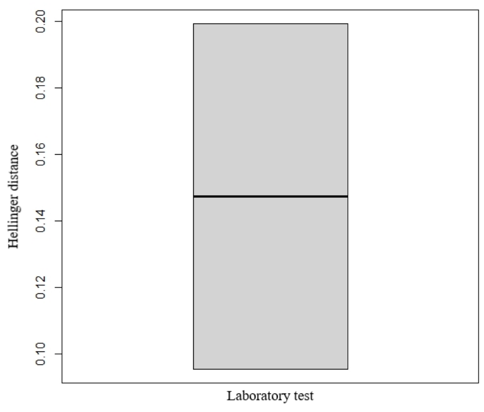


Figure 19: The boxplot shows the variation of Hellinger distance between the original and HMC's synthetic categorical variables.

## Section S11: Results Plot

The following is an outcome of the GCP decomposition with Gaussian loss function, and using HMC for the patient factor matrix simulation of categorical variables.

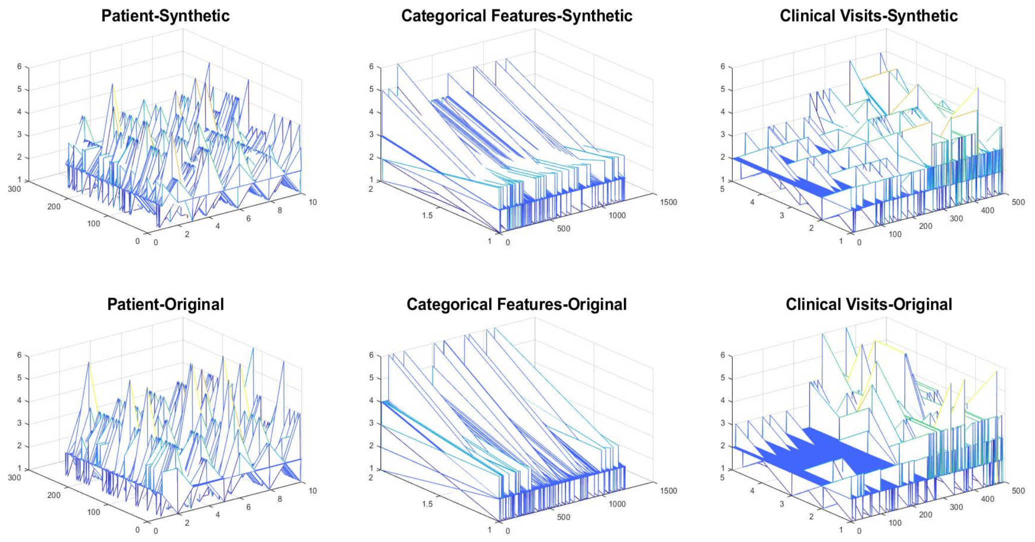


Figure 20: The different modes (Patients, Categorical features, and Clinical visits) of HMC's generated categorical data are shown.

## References

[1] Hong D, Kolda TG, Duersch JA. Generalized canonical polyadic tensor decomposition. SIAM Review. 2020;62(1):133-163.
